# Guidelines are needed for studies of pre-treatment HIV drug resistance: a methodological study

**DOI:** 10.1186/s12874-021-01258-1

**Published:** 2021-04-19

**Authors:** Lawrence Mbuagbaw, Clémence Ongolo-Zogo, Olivia C. Mendoza, Babalwa Zani, Frederick Morfaw, Agatha Nyambi, Annie Wang, Michel Kiflen, Hussein El-Kechen, Alvin Leenus, Mark Youssef, Nadia Rehman, Lucas Hermans, Virginia MacDonald, Silvia Bertagnolio

**Affiliations:** 1 Department of Health Research Methods, Evidence and Impact, McMaster University, Hamilton, ON Canada; 2 Biostatistics Unit, Father Sean O’Sullivan Research Centre, St Joseph’s Healthcare, Hamilton, ON Canada; 3 Centre for Development of Best Practices in Health (CDBPH), Yaoundé Central Hospital, Yaoundé, Cameroon; 4 McMaster Health Forum, Hamilton, ON Canada; 5 Faculty of Health Sciences, McMaster University, Hamilton, ON Canada; 6 Knowledge Translation Unit, University of Cape Town Lung Institute, Cape Town, South Africa; 7 Department of Obstetrics & Gynecology, McMaster University, Hamilton, ON Canada; 8 Ontario HIV Treatment Network, Toronto, ON Canada; 9 Population Health Research Institute, Hamilton, ON Canada; 10 School of Medicine, University of Ottawa, Ottawa, ON Canada; 11 Virology, Department of Medical Microbiology, University Medical Center Utrecht, Utrecht, The Netherlands; 12 Wits Reproductive Health and HIV Institute, University of the Witwatersrand, Johannesburg, South Africa; 13 Department of HIV, Hepatitis, and Sexually Transmitted Diseases, World Health Organization, Geneva, Switzerland

**Keywords:** HIV, Pre-treatment drug resistance, Reporting, Guidelines

## Abstract

**Background:**

The expansion of access to antiretroviral therapy (ART) has been accompanied by an increase in pre-treatment drug resistance (PDR). While it is critical to monitor the increasing prevalence of PDR across countries and populations to inform optimal regimen selection, the completeness of reporting is often suboptimal, limiting the interpretation and generalizability of the results. Indeed, there is no formal guidance on how studies investigating the prevalence of drug resistance should be reported. Thus, we sought to determine the completeness of reporting in studies of PDR and the factors associated with sub-optimal reporting to ascertain the need for guidelines.

**Methods:**

As part of a systematic review on the global prevalence of PDR in key populations (men who have sex with men, sex workers, transgender people, people who inject drugs and people in prisons), we searched 10 electronic databases until January 2019. We extracted information on selected study characteristics useful for interpreting prevalence data. Data were extracted in duplicate. Analyses of variance and correlation were used to explore factors that may explain the number of items reported.

**Results:**

We found 650 studies of which 387 were screened as full text and 234 were deemed eligible. The included studies were published between 1997 and 2019 and included a median of 239 (quartile 1 = 101; quartile 3 = 778) participants. Most studies originated from high-income countries (125/234; 53.0%). Of 23 relevant data items, including study design, setting, participant sociodemographic characteristics, HIV risk factors, type of resistance test conducted, definition of resistance, the mean (standard deviation) number of items reported was 13 (2.2). We found that more items were reported in studies published more recently (*r =* 0.20; *p* < 0.002) and in studies at low risk of bias (F [2231] = 8.142; *p* < 0.001).

**Conclusions:**

Incomplete reporting in studies on PDR makes characterising levels of PDR in subpopulations across countries challenging. Hence, guidelines are needed to define a minimum set of variables to be included in such studies.

**Supplementary Information:**

The online version contains supplementary material available at 10.1186/s12874-021-01258-1.

## Background

An estimated 37.9 million people were living with HIV worldwide in 2018 [1]. While HIV incidence has decreased over the years, the large number of people living with HIV can be attributed to improvements in the management of HIV infection by early detection and early treatment with antiretroviral therapy (ART). One major hindrance to the effectiveness of ART is drug resistance, as it limits the number of effective drugs, increases the potential for onward transmission, and compromises survival [2, 3].

Drug resistance to ART may be acquired when there is viral replication in the presence of a drug. This is often due to suboptimal adherence to medication. In some individuals, drug resistant viral strains are already present prior to ART initiation. This is referred to as pre-treatment drug resistance (PDR). PDR can be due to infection with a drug resistant viral strain, also referred to as transmitted drug resistance, or due to prior exposure to antiretroviral treatment (e.g., women and children exposed to treatment as part of prevention of vertical transmission programs and people who abandoned prior treatments).

PDR is a recognised global health problem [4]. People with PDR are more likely to have treatment failure, to discontinue treatment, and to develop new drug resistant strains [5]. The rise in drug resistance is one of the greatest threats to global health, and without urgent attention can result in millions of deaths, an increase in new harder-to-treat strains of HIV and higher healthcare costs [6]. The prevalence of HIV PDR varies worldwide, and it can be as high as 25% in some countries [7], likely due to the efforts to expand widespread availability of ART in these countries. PDR is concerning because it can exist among people who are unaware of their HIV infection, and they may unknowingly transmit resistant virus to others. Understanding the levels of PDR is of importance to researchers, clinicians, and policymakers because this information can inform guidelines on how treatment should be tailored and what drugs should be used as first-line treatments.

In high-income countries where selection of ART is individualised, drug resistance testing is performed prior to initiating ART [8]. On the other hand, in low- and middle-income countries following the public health approach to HIV treatment and care [5], selection of first-line treatment is not informed by individual drug resistance tests, but rather by population-based surveys of pre-treatment drug resistance. Typically, ART regimens include a combination of three antiretroviral drugs (ARVs) belonging to two different drug classes, one anchor drug (e.g., from the non-nucleoside reverse transcriptase drug class, or NNRTI) and two backbone drugs (from nucleoside reverse transcriptase drug class, or NRTI). Currently, the World Health Organisation (WHO) recommends moving away from regimens using NNRTIs as the anchor drug in first-line treatment if the prevalence of PDR to that drug class is ≥10% [5].

Effective and timely response to high levels of PDR also requires monitoring resistance emerging in sub-populations, such as key populations (men who have sex with men, sex workers, transgender people, people who inject drugs and people in prisons), pregnant women, adolescents and children, acknowledging that levels of resistance may vary by sex and ethnicity (due to different ART exposures) and HIV subtype [9–12].

Unfortunately, adequate monitoring of PDR across countries and populations is often challenged by heterogenous and inadequate data reporting. This implies that studies that collect information on drug resistance should be designed and reported appropriately. In other words, the prevalence of drug resistance should be interpreted with due consideration of the precision of the estimates, the representativeness and diversity of the participants included, the techniques used to measure resistance, the participants’ transmission risk group, prior exposure to treatments and class of drug for which resistance was tested. While some of these concerns are relevant to all studies of prevalence, many are unique to HIV drug resistance.

The purpose of this work is to provide evidence to inform the development of guidelines for reporting studies of HIV drug resistance. In this paper we investigated the completeness of reporting of studies reporting the prevalence of PDR.

## Methods

### Design

As part of a systematic review on the global prevalence of PDR in key populations living with HIV [13, 14], we conducted a separate methodological study on reporting completeness.

### Data sources

We searched PubMed, Scopus, CINAHL, LILACS, WHO Global Health Libraries, Ovid Global Health, Sociological Abstracts, PsycINFO, EMBASE, and POPLINE from inception to January 2019 (See Additional File 1 for search strategy).

### Eligibility

We included studies of any design, reporting PDR and published in full text. Eligible studies were those that reported the number of people tested for drug resistance and the number with resistance mutations from one of the following key populations: people who inject drugs, men who have sex with men, transgender people, sex workers or people in prisons. We excluded abstracts because they are unlikely to report all relevant items. Modelling studies were not eligible.

### Data extraction and management

Screening and data extraction were performed using DistillerSR (Evidence Partners, Ottawa, Canada). Duplicates (same study identified from multiple databases) were identified and removed using the “duplicate detection” function of DistillerSR and during full text screening. We extracted basic bibliometric information such as: author name, year of publication, country of study i.e., the country in which the participants were recruited (organised by region and income level). Region was determined based on the WHO regional groupings of countries [15], and income level was determined based on the World Bank classification [16]. We collected study characteristics such as sample size, design, location, setting, source of funding and whether the studies performed a sample size calculation.

We checked for the reporting of baseline characteristics such as: age, gender, sexual orientation, transmission risk group, profession, country of residence, ethnicity, education, income level and prior exposure to ART.

We also assessed the availability and completeness of the following information on drug resistance including: the type of resistance testing used (such as population based-sequencing or Sanger vs next generation sequencing); the number of participants enrolled as well as the number of available genotypes; the drug classes for which resistance testing was conducted; the definition and interpretation of drug resistance and whether the authors distinguished between major (greater reductions in drug susceptibility) and minor drug resistance mutations. We also extracted data on source of funding.

### Assessment of risk of bias

We assessed the risk of bias in the reporting of prevalence using an adapted version of a tool proposed by Hoy et al. [17] Using this tool, risk of bias is based on the representativeness of the sample, the sampling frame, sampling technique, response bias, the use of proxies, case definition, validity of measurements, uniformity of data collection, the prevalence period and the appropriateness of the numerator and denominator. We judged each study’s risk of bias as overall high, low, or moderate based on an appraisal of these items. For instance, a judgment of high risk of bias would imply that further research is very likely to have an important impact on our confidence in the estimate of prevalence and is likely to change the estimate. Moderate risk of bias would imply that further research is likely to have an important impact on our confidence in the estimate of prevalence and may change the estimate and low risk of bias would imply that further research is very unlikely to change our confidence in the estimate of prevalence.

All data were extracted in duplicate by pairs of reviewers (OM, COZ, BZ, FM, AN, AW, MK, HE, AL, MY, NR) and disagreements were adjudicated by a third reviewer (LM). Agreement was computed separately for data extraction and risk of bias using the Kappa statistic [18], since we used a tool that has not been previously validated for prevalence studies of drug resistance.

### Data analyses

Our findings are reported as counts and percentages and mean (standard deviation) or median (quartile 1; quartile 3) as appropriate. We created a summary score for the number of items reported (possible range 0–23). We examined categorical factors that may be associated with reporting completeness using one-way Analysis of Variance (ANOVA) and Tukey’s test for post-hoc analysis (source of funding, income level, region). We also examined the correlation between the number of reported items and year and sample size, using Pearson’s correlation coefficient. These factors have been shown to be associated with reporting [19]. For these analyses, studies that reported on more than one country were excluded when they had overlapping income levels and regions. F-tests, degrees of freedom, Pearson’s correlation coefficient and *p*-values are reported. We used the number of items reported as measure of completeness of reporting.

## Results

**Fig. 1:**
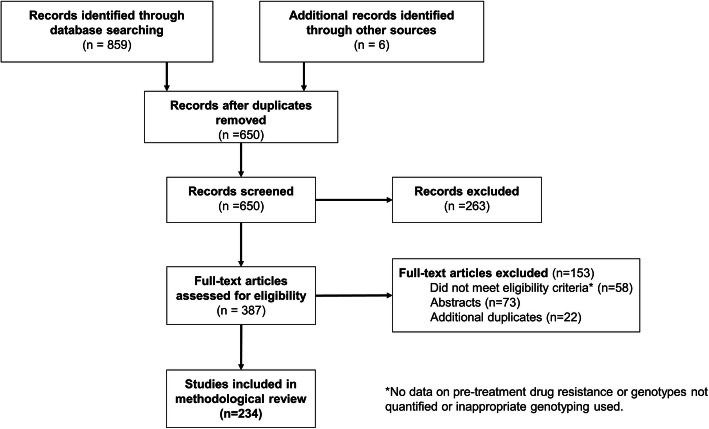
Study selection flow diagram. The flow diagram details the search and selection process applied during the review

Our searches retrieved 865 studies of which 215 were duplicates, leaving 650. After screening titles and abstracts, 263 were excluded and 387 were screened as full text. Only 234 were eligible (See Additional File 2). Agreement on data extraction was almost perfect (91.0%). Agreement on the adapted risk of bias tool was moderate (60.0%) [18]. Study screening and selection is shown in a flow diagram. See Fig. 1.

The included studies were published between 1997 and 2019 and included a median (quartile 1; quartile 3) of 239 (101;778) participants. Most studies were from high-income countries (125/239; 53.0%), and from the European region (82/239;35.0%) or the American region (68/239; 29.1%). Further details are provided in Table 1.

**Table 1:** Characteristics of included studies

Variable	Statistic (***N*** = 234)
**Year of publication: median (q1; q3)**	2013 (2009;2015)
**Sample size: median (q1; q3)**	239 (101;778)
**Income: n (%)**	
High	125 (53.4)
Upper middle	86 (36.8)
Lower middle	16 (6.8)
Low	3 (1.3)
Multiple	4 (1.7)
**Region: n (%)**	
Africa	7 (3.0)
America	68 (29.1)
Eastern Mediterranean	4 (1.7)
Europe	82 (35.0)
South East Asia	12 (5.1)
Western Pacific	56 (23.9)
Multiple^a^	5 (2.1)
**Funding: n (%)**	
Not reported	121 (51.7)
Government	51 (21.8)
Multiple^a^	3 (1.3)
Industry	19 (8.1)
Private	40 (17.1)

^a^Belonging to more than one category

### Reporting completeness

Out of 23 possible items, the mean (standard deviation) number of items reported was 13 (2.2). Table 2 outlines the number of studies that reported each item.

**Table 2:** Proportion of data items reported

#	Item	n (%)
**Study level data**		
1	Setting of study, e.g. hospital, community, prison etc.	132 (56.4)
2	Location of study, e.g. country, city, village	230 (98.3)
3	Study design, e.g. cross-sectional, retrospective etc.	103 (44.0)
4	Sample size justification, i.e. (was the sample size justified?)	6 (2.6)
**Participant data**		
5	Age	186 (79.5)
6	Sex/Gender	203 (86.8)
7	Sexual orientation	179 (76.5)
8	Transmission risk group, e.g. injections drug use	178 (76.1)
9	Profession	9 (3.8)
10	Place of residence, e.g. urban, rural	33 (14.1)
11	Ethnicity	83 (35.5)
12	Level of education	19 (8.1)
13	Income	6 (2.6)
14	Exposure to antiretroviral therapy, e.g. treatment-naïve	216 (92.3)
**Information on resistance testing**		
15	Type of resistance test, e.g. Sanger sequencing, next generation sequencing	199 (85.0)
16	Mutation list used, e.g. ^a^WHO SDRM list	204 (87.2)
17	Number of genotypes (as opposed to the number of participants)	134 (57.3)
18	Resistance to NNRTI drug class	222 (94.9)
19	Resistance to NRTI drug class	220 (94.0)
20	Resistance to PI drug class	207 (88.5)
21	Resistance to INSTI drug class	5 (2.1)
22	Clinical Relevance, e.g. mutations associated with reduced virological response	52 (22.2)
**Other information**		
23	Source of funding	195 (83.3)

*NNRTI* Non-Nucleoside Reverse Transcriptase*, NRTI* Nucleoside Reverse Transcriptase Inhibitors*, PI* Protease Inhibitors*, INSTI* Integrase Strand Transfer Inhibitor*,* ^a^ *WHO SDRM* World Health Organisation Surveillance Drug Resistance Mutation

### Risk of bias

**Fig. 2:**
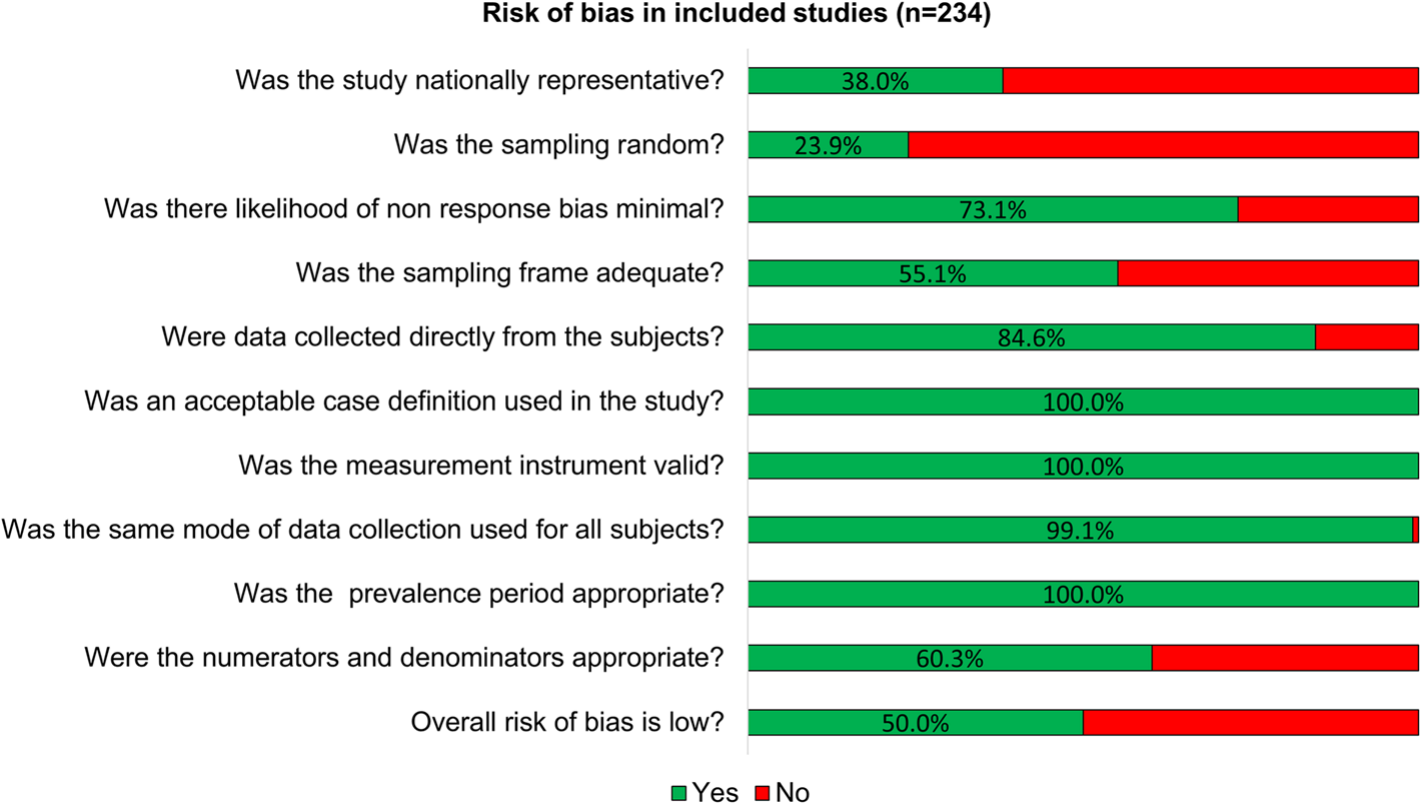
Summary of risk of bias assessments. Risk of bias assessment for ten predefined domains for each included study

Of the 234 included studies, 117 (50.0%) were at low risk of bias, 52 (22.2%) at moderate risk of bias and 65 (27.8%) at high risk of bias. The three most frequent concerns were related to sampling methods, i.e., only 89 (38.0%) studies reported a nationally representative sampling method; 56 (23.9%) reported the use of random sampling approaches and 129 (55.1%) reported adequate sampling frames i.e., a list of people forming the population from which the sample is taken. Risk of bias is summarised in Fig. 2**.**

#### Factors associated with completeness of reporting

The mean number of items reported differed across regions (F [5223] =2.663; *p* = 0.023). Post hoc analysis revealed a difference between Europe and the Western Pacific region (Mean difference [MD] = − 1.22; 9% CI − 0.12 to − 2.30). There was no difference in the mean number of items reported by source of funding (F [3190] = 0.801;*p* = 0.495) or by income level (F [3226] = 1.331; *p* = 0.265). The mean number of items differed by risk of bias (F [2231] =8.142; *p* < 0.001), with significant differences between studies at high risk of bias and the studies at low risk (MD -1.29; 95% CI − 0.21 to − 0.51; *p <* 0.001) or moderate risk of bias (MD -1.17; 95% CI − 2.11 to − 0.23; *p* = 0.010), based on post-hoc analyses. There was a positive correlation between number of items reported and year of publication (*r =* 0.20; *p* < 0.002) but not sample size (*r =* 0.05; *p* = 0.366). These results are displayed in additional file 4.

## Discussion

We found that many key features of studies on the prevalence of PDR were not reported in a large proportion of studies assessed, and that year of publication and risk of bias might explain the differences in the number of items reported. This may be for several reasons. First, there is currently no existing guidance on standard reporting of PDR data and how this information should be reported and therefore authors would tailor their results to their objectives and audiences. For example, if authors do not plan to examine the role of certain variables on their prevalence estimates, these variables may not be collected. Second, studies published before the value of investigating PDR in key population was recognised might have been less likely to report disaggregated findings. Third, studies conducted using laboratory databases may not have captured sociodemographic information.

These findings are not surprising, as it has been shown in other fields that the completeness of reporting is higher in more recently published studies [20, 21]. Some studies have found links between certain risk of bias items and reporting completeness in trials [22, 23]. Furthermore, studies that are inadequately reported may also omit key information required to judge risk of bias and would therefore be judged to be at a high risk of bias. Region, income level, source of funding and sample size were not associated with completeness of reporting in this study even though they have been shown to be associated with reporting completeness in other studies [23].

The reliability of prevalence estimates is highly contingent on the sample size. While a large sample size does not necessarily eliminate other sources of bias, it improves precision. As such it may be of interest to see how the number of participants to be included in the study was determined. It is expected that a larger sample size will be needed if the prevalence is low [24]. The type of test and mutation list used may help to ensure that studies are comparable and appropriately interpretable, and to explain heterogeneity across studies. For example, some discrepancies exist between the various genotypic testing procedures and algorithms for interpretation [25, 26]. More so, the mutation lists are often updated, suggesting that results may differ over time with the same list as it gets modified [27]. Given that not all sequencing attempts are successful, it is important for researchers to distinguish between the number of participants included in the study and the number of genotypes successfully sequenced. The drug class for which resistance mutations are sought should also be clearly reported to allow adequate interpretation of the findings; unfortunately, in many instances a generic PDR prevalence is reported, without disaggregation of the results by drug and/or drug class. Not all mutations confer the same level of resistance to ART and therefore it is important that “major” mutations are distinguished from “minor” ones, so that the prevalence is not unduly inflated [28]. Finally, source of funding helps to identify potential conflicts of interest in research and should be reported in all manuscripts.

This work is not without limitations. First, this work is based on a systematic review on the prevalence of PDR in KPs [13]. While it is unlikely that studies of acquired drug resistance would be reported differently it is important to note that our findings may not be generalizable to all types of HIV drug resistance. Second, to the best of our knowledge, our adapted tool for risk of bias has never been used for studies of HIV drug resistance. We noted that agreement was moderate, indicating that the use of this tool and the included items could be better adapted for HIV drug resistance studies. Some items did not have a particularly good ability to discriminate between studies at high or low risk of bias. For example, in all studies, the case definitions were acceptable, and tools used for detecting drug resistance were valid. Further psychometric evaluation of this tool is warranted in studies of prevalence of HIV drug resistance. As such, we recommend caution in the interpretation of these results based on a preliminary list of items and an imperfect risk of bias tool.

This work is meant to inform the development of guidelines on studies reporting the prevalence of drug resistance mutations and to enhance the quality of systematic reviews of such studies. Pending a formal appraisal and selection of preferred items to be reported (guideline development is ongoing: https://www.equator-network.org/library/reporting-guidelines-under-development/reporting-guidelines-under-development-for-observational-studies/#CEDRIC), the items addressed in this paper have face validity. The setting, location and design of every study is relevant to readers. The sociodemographic characteristics such as age, gender, ethnicity, sexual orientation, transmission risk group, profession, place of residence, ethnicity, level of education and income allow readers to adequately characterise the population and describe inequities in health. More so, many of these factors have been shown to be associated with different levels of PDR or transmitted drug resistance [29–33]. Further to the items above, researchers may also be interested in pooling data from the individual mutations. An appropriate framework for how these should be reported is beyond the scope of this work.

The future steps needed are to seek consensus on a list of key reporting items and provide guidance on how they should be reported, with appropriate justification for why they are needed.

## Conclusion

The completeness of reporting in studies of HIV PDR prevalence is low. Even though reporting has improved over time, guidance is needed to ensure complete and uniform reporting, thus improving appropriate interpretation, generalizability and comparability of prevalence estimates, accounting for differences in geographical settings and populations. Tailored tools may be required to appraise risk of bias issues that are specific to studies of HIV drug resistance.

## Supplementary Information

**Additional file 1.** Search Strategy. Search terms and strategy used to identify relevant articles in electronic databases

**Additional file 2.** Included studies. List of included studies

**Additional file 3.** PRISMA Checklist

**Additional file 4.** Bar charts and scatterplots

## Data Availability

Not applicable.

## Abbreviations

ART: Antiretroviral therapy
ARV: Antiretroviral
HIV: Human immunodeficiency virus
KP: Key population
PDR: Pre-treatment drug resistance
WHO: World Health Organisation
