# Supplementary material for: Guidelines are needed for studies of pre-treatment HIV drug resistance: a methodological study
Source: BMC Med Res Methodol. 2021 Apr 19;21:76. doi: 10.1186/s12874-021-01258-1 (PMC8056637; doi:10.1186/s12874-021-01258-1)
Supplement: Supplementary file 1 — Additional file 1. Search Strategy. Search terms and strategy used to identify relevant articles in electronic databases [file 12874_2021_1258_MOESM1_ESM.docx]

**SEARCH STRATEGY**

The following 10 electronic databases were searched for papers published up until January 2019 that included the pre-defined search terms described below: PubMed, Scopus, WHO Global Health Libraries, Ovid Global Health, Sociological Abstracts, PsycINFO, EMBASE, and POPLINE. Secondary reference searching was conducted on all studies included in the review. Further, selected experts in the field were contacted to identify additional articles not identified through other search methods. Search terms related to HIV, drug resistance, HIV treatments and key populations were used to develop a search strategy.

**1: HIV/AIDS**

“HIV Infections” [MeSH] OR “HIV”[MeSH] OR “hiv”[tw] OR “hiv-1”[tw] OR “hiv-2”[tw] OR “hiv1”[tw] OR “hiv2”[tw] OR hiv infect*[tw] OR “human immunodeficiency virus”[tw] OR “human immunedeficiency virus”[tw] OR “human immuno-deficiency virus”[tw] OR “human immune-deficiency virus”[tw] OR ((human immun*) AND (“deficiency virus”[tw])) OR “acquired immunodeficiency syndrome”[tw] OR “acquired immunedeficiency syndrome”[tw] OR “acquired immuno-deficiency syndrome”[tw] OR “acquired immune-deficiency syndrome”[tw] OR ((acquired immun*) AND (“deficiency syndrome”[tw])) OR "Sexually Transmitted Diseases, Viral"[MeSH:NoExp]

**2: Drug resistance/Treatment failure**

"Drug Resistance"[Mesh] OR resistance[tw] OR “drug resistance”[tw] OR “drug resistant”[tw] OR resistant[tw] OR mutant[tw] OR mutation[tw] OR genotype[tw] OR genotyp*[tw] OR genotypic[tw] OR “virological failure”[tw] OR “treatment failure”[tw] OR "Treatment Failure"[Mesh] OR "Viral Load"[Mesh] OR “viral load” OR “transmitted resistance”[tw] or “pre-treatment drug resistance”[tw] OR naïve[tw] OR “transmitted drug resistance”[tw] OR “TDR” [tw]

**3: HIV treatment**

"Anti-HIV Agents"[Mesh] OR “ARV” or “ART” or “HIV treatment” OR “Antiretroviral” OR atazanavir OR darunavir OR dolutegravir OR fosamprenavir OR indinavir OR lopinavir OR saquinavir OR efavirenz OR enfuvirtide OR etravirine OR lamivudine OR maraviroc OR nevirapine OR raltegravir OR rilpivirine OR saquinavir OR tenofovir OR tipranavir OR “non-nucleoside reverse-transcriptase inhibitors” OR “NNRTI” OR “nucleoside reverse-transcriptase inhibitors” OR “NRTI” OR “HAART” OR “protease inhibitor” OR “boosted protease inhibitor” OR “PI” OR “bPI” OR “highly active antiretroviral therapy” OR “antiretroviral therapy” OR stavudine OR zidovudine OR nevirapine OR efavirenz OR antiretrovir*

**4: Key populations**

1. **Men who have sex with men and transgender**

“men who have sex with men”[ALL] OR "Homosexuality"[Mesh] OR bisexuality[Mesh] OR queer[ALL] OR “men-who-have-sex-with-men”[ALL] OR “MSM”[ALL] OR “gay”[ALL] OR “homosexual”[ALL] OR “homosexuals”[ALL] OR “bi-sexual”[ALL] OR LGB*[ALL] OR "Transgender Persons"[Mesh] OR transgender[ALL] OR transexualism[ALL] OR LBG*[ALL] OR “sexual minority”[TW] OR transsexual[ALL] OR “sexual orientation”[ALL]

1. **Sex workers**

"Prostitution"[Mesh] OR “sex worker”[ALL] OR “sex workers”[ALL] OR “commercial sex worker”[ALL] OR “ prostitute”[ALL] OR brothel*[ALL] OR escort[ALL] OR “commercial sex”[ALL] OR “ commercial sex worker”[ALL] OR “sex work”[ALL] OR prostitut*[ALL] OR prostitution[ALL] OR brothel*[ALL] OR escort[ALL]

1. **People who inject drugs**

“drug user”[ALL] OR “drug addict”[ALL] OR addict[ALL] OR “injecting drug user”[ALL] OR “injection drug user”[ALL] OR “injecting drug users”[ALL] OR “injection drug users”[ALL] OR “people who inject drugs”[ALL] OR “people who use drugs”[ALL] OR “substance abuse”[ALL] OR “substance misuse”[ALL] OR “substance use”[ALL] OR “substance use disorder”[ALL] OR “substance user[ALL] OR “PWID”[ALL] OR "Substance Abuse, Intravenous"[Mesh] OR addiction[ALL]

1. **Prisoners and other people in closed settings**

"Prisoners"[Mesh] AND "Prisons"[Mesh] OR “forced treatment”[ALL] OR “compulsory treatment”[ALL] OR detention[ALL] OR “mandatory detention”[ALL] OR “detention centre”[ALL] OR “detention center”[ALL] OR prison[ALL] OR prisoner[ALL] OR offender[ALL] OR jail[ALL] OR correctional[ALL] OR inmate*[ALL]
