# Supplementary material for: Guidelines are needed for studies of pre-treatment HIV drug resistance: a methodological study
Source: BMC Med Res Methodol. 2021 Apr 19;21:76. doi: 10.1186/s12874-021-01258-1 (PMC8056637; doi:10.1186/s12874-021-01258-1)
Supplement: Supplementary file 2 — Additional file 2. Included studies. List of included studies [file 12874_2021_1258_MOESM2_ESM.docx]

**List of included studies**

1. Alexander CS, Dong W, Schechter MT, O'Shaughnessy MV, Strathdee SA, Mo T, Montaner JS, Harrigan PR: **Prevalence of primary HIV drug resistance among seroconverters during an explosive outbreak of HIV infection among injecting drug users**. *AIDS* 1999, **13**(8):981-985.

2. Alexiev I, Shankar A, Dimitrova R, Gancheva A, Kostadinova A, Teoharov P, Golkocheva E, Nikolova M, Muhtarova M, Elenkov I *et al*: **Origin and spread of HIV-1 in persons who inject drugs in Bulgaria**. *Infect Genet Evol* 2016, **46**:269-278.

3. Alpsar D, Agacfidan A, Lubke N, Verheyen J, Eraksoy H, Cagatay A, Bozkaya E, Kaiser R, Akgul B: **Molecular epidemiology of HIV in a cohort of men having sex with men from Istanbul**. *Med Microbiol Immunol* 2013, **202**(3):251-255.

4. Amaral AG, Oliveira IB, Carneiro DC, Alcantara LC, Monteiro-Cunha JP: **An overview of the molecular and epidemiological features of HIV-1 infection in two major cities of Bahia state, Brazil**. *Mem Inst Oswaldo Cruz* 2017, **112**(6):411-418.

5. Ananworanich J, Phanuphak N, de Souza M, Paris R, Arroyo M, Trichavaroj R, Sirivichayakul S, Shikuma C, Phanuphak P, Kim JH *et al*: **Incidence and characterization of acute HIV-1 infection in a high-risk Thai population**. *J Acquir Immune Defic Syndr* 2008, **49**(2):151-155.

6. Ananworanich J, Sirivichayakul S, Pinyakorn S, Crowell TA, Trichavaroj R, Weerayingyong J, Chomchey N, Fletcher JL, van Griensven F, Phanuphak P *et al*: **High prevalence of transmitted drug resistance in acute HIV-infected Thai men who have sex with men**. *J Acquir Immune Defic Syndr* 2015, **68**(4):481-485.

7. Andersson E, Nordquist A, Esbjornsson J, Flamholc L, Gisslen M, Hejdeman B, Marrone G, Norrgren H, Svedhem V, Wendahl S *et al*: **Increase in transmitted drug resistance in migrants from sub-Saharan Africa diagnosed with HIV-1 in Sweden**. *AIDS* 2018, **32**(7):877-884.

8. Andreani GE, C.; Ceballos, A.; Ambrosioni, J.; Petroni, A.; Pugliese, D.; Bouzas, MB.; Giuliano, SF.; Weissenbacher, MC.; Losso, M.; Benetucci, J.: **Detection of HIV-1 dual infections in highly exposed treated patients**. *Virology Journal* 2011, **8**.

9. Ariffin TA, Mohamad S, Yusuf WN, Shueb RH: **Antiretroviral drug resistance and HIV-1 subtypes among treatment-naive prisoners in Kelantan, Malaysia**. *J Infect Dev Ctries* 2014, **8**(8):1063-1067.

10. Audelin AM, Gerstoft J, Obel N, Mathiesen L, Laursen A, Pedersen C, Nielsen H, Jensen J, Nielsen L, Nielsen C *et al*: **Molecular phylogenetics of transmitted drug resistance in newly diagnosed HIV Type 1 individuals in Denmark: a nation-wide study**. *AIDS Res Hum Retroviruses* 2011, **27**(12):1283-1290.

11. Avidor B, Turner D, Mor Z, Chalom S, Riesenberg K, Shahar E, Pollack S, Elbirt D, Sthoeger Z, Maayan S *et al*: **Transmission patterns of HIV-subtypes A/AE versus B: inferring risk-behavior trends and treatment-efficacy limitations from viral genotypic data obtained prior to and during antiretroviral therapy**. *PLoS One* 2013, **8**(3):e57789.

12. Avila-Rios S, Garcia-Morales C, Matias-Florentino M, Romero-Mora KA, Tapia-Trejo D, Quiroz-Morales VS, Reyes-Gopar H, Ji H, Sandstrom P, Casillas-Rodriguez J *et al*: **Pretreatment HIV-drug resistance in Mexico and its impact on the effectiveness of first-line antiretroviral therapy: a nationally representative 2015 WHO survey**. *The lancet HIV* 2016, **3**(12):e579-e591.

13. Avila-Rios S, Mejia-Villatoro CR, Garcia-Morales C, Soto-Nava M, Escobar I, Mendizabal R, Giron A, Garcia L, Reyes-Teran G: **Prevalence and patterns of HIV transmitted drug resistance in Guatemala**. *Revista panamericana de salud publica = Pan American journal of public health* 2011, **30**(6):641-648.

14. Babic DZ, Zelnikar M, Seme K, Vandamme AM, Snoeck J, Tomazic J, Vidmar L, Karner P, Poljak M: **Prevalence of antiretroviral drug resistance mutations and HIV-1 non-B subtypes in newly diagnosed drug-naive patients in Slovenia, 2000-2004**. *Virus Res* 2006, **118**(1-2):156-163.

15. Bannister WP, Cozzi-Lepri A, Clotet B, Mocroft A, Kjaer J, Reiss P, von Wyl V, Lazzarin A, Katlama C, Phillips AN *et al*: **Transmitted drug resistant HIV-1 and association with virologic and CD4 cell count response to combination antiretroviral therapy in the EuroSIDA Study**. *J Acquir Immune Defic Syndr* 2008, **48**(3):324-333.

16. Bermudez A, E H, L. R. F S, A A, De M, M B, R S, M. D C, C S, De B *et al*: **Antiretroviral drug resistance in a respondent-driven sample of HIV-infected men who have sex with men in Brazil**. *Journal of Acquired Immune Deficiency Syndromes* 2011, **57**:S186-S192.

17. Bes M, Piron M, Casamitjana N, Gregori J, Esteban JI, Ribera E, Quer J, Puig L, Sauleda S: **Epidemiological trends of HIV-1 infection in blood donors from Catalonia, Spain (2005-2014)**. *Transfusion* 2017, **57**(9):2164-2173.

18. Bezemer D, Jurriaans S, Prins M, van der Hoek L, Prins JM, de Wolf F, Berkhout B, Coutinho R, Back NK: **Declining trend in transmission of drug-resistant HIV-1 in Amsterdam**. *AIDS* 2004, **18**(11):1571-1577.

19. Bhusal N, Sutthent R, Horthongkham N, Athipanyasilp N, Kantakamalakul W: **Prevalence of HIV-1 Subtypes and Antiretroviral Drug Resistance Mutations in Nepal**. *Curr HIV Res* 2016, **14**(6):517-524.

20. Boden D, Hurley A, Zhang L, Cao Y, Guo Y, Jones E, Tsay J, Ip J, Farthing C, Limoli K *et al*: **HIV-1 drug resistance in newly infected individuals**. *JAMA* 1999, **282**(12):1135-1141.

21. Bontell I, Cuong do D, Agneskog E, Diwan V, Larsson M, Sonnerborg A: **Transmitted drug resistance and phylogenetic analysis of HIV CRF01_AE in Northern Vietnam**. *Infect Genet Evol* 2012, **12**(2):448-452.

22. Bonura F, Tramuto F, Vitale F, Perna AM, Viviano E, Romano N, Group for HIVASiS: **Transmission of drug-resistant HIV type 1 strains in HAART-naive patients: a 5-year retrospective study in Sicily, Italy**. *AIDS Res Hum Retroviruses* 2010, **26**(9):961-965.

23. Booth CL, Garcia-Diaz AM, Youle MS, Johnson MA, Phillips A, Geretti AM: **Prevalence and predictors of antiretroviral drug resistance in newly diagnosed HIV-1 infection**. *J Antimicrob Chemother* 2007, **59**(3):517-524.

24. Brindeiro RD, RS.; Sabino, EC.; Morgado, MG.; Pires, IL.; Brigido, L.; Dantas, MC.; Barreira, D.; Teixeira, PR.; Tanuri, A: **Brazilian Network for HIV Drug Resistance Surveillance (HIV-BResNet): A survey of chronically infected individuals**. *AIDS* 2003, **17**(7):1063-1069.

25. Briones C, Perez-Olmeda M, Rodriguez C, del Romero J, Hertogs K, Soriano V: **Primary genotypic and phenotypic HIV-1 drug resistance in recent seroconverters in Madrid**. *J Acquir Immune Defic Syndr* 2001, **26**(2):145-150.

26. Brooks JI, Niznick H, Ofner M, Merks H, Angel JB: **Local phylogenetic analysis identifies distinct trends in transmitted HIV drug resistance: implications for public health interventions**. *BMC Infect Dis* 2013, **13**:509.

27. Buchacz K, Young B, Palella FJ, Jr., Armon C, Brooks JT, investigators HIVOS, investigators HIVOSH: **Trends in use of genotypic resistance testing and frequency of major drug resistance among antiretroviral-naive persons in the HIV Outpatient Study, 1999-2011**. *J Antimicrob Chemother* 2015, **70**(8):2337-2346.

28. Budambula V, Musumba FO, Webale MK, Kahiga TM, Ongecha-Owuor F, Kiarie JN, Sowayi GA, Ahmed AA, Ouma C, Were T: **HIV-1 protease inhibitor drug resistance in Kenyan antiretroviral treatment-naive and -experienced injection drug users and non-drug users**. *AIDS Res Ther* 2015, **12**:27.

29. Cardoso LP, Queiroz BB, Stefani MM: **HIV-1 pol phylogenetic diversity and antiretroviral resistance mutations in treatment naive patients from Central West Brazil**. *J Clin Virol* 2009, **46**(2):134-139.

30. Carobene M, Bolcic F, Farias MS, Quarleri J, Avila MM: **HIV, HBV, and HCV molecular epidemiology among trans (transvestites, transsexuals, and transgender) sex workers in Argentina**. *J Med Virol* 2014, **86**(1):64-70.

31. Carras T: **A multicenter study of initiation of antiretroviral therapy and transmitted drug resistance in antiretroviral-naive adolescents and young adults with HIV in New York City**. *Clinical Infectious Diseases* 2014, **58**(6):865-872.

32. Carvalho BC, Cardoso LP, Damasceno S, Stefani MM: **Moderate prevalence of transmitted drug resistance and interiorization of HIV type 1 subtype C in the inland North State of Tocantins, Brazil**. *AIDS Res Hum Retroviruses* 2011, **27**(10):1081-1087.

33. Cavalcanti AM, Brito AM, Salustiano DM, Lima KO, Silva SP, Diaz RS, Lacerda HR: **Primary resistance of HIV to antiretrovirals among individuals recently diagnosed at voluntary counselling and testing centres in the metropolitan region of Recife, Pernambuco**. *Mem Inst Oswaldo Cruz* 2012, **107**(4):450-457.

34. Ceccherini S, S A, C F, M R, De C, M S, Di C, De F, S A: **Characterization of the patterns of drug-resistance mutations in newly diagnosed HIV-1 infected patients naïve to the antiretroviral drugs**. *BMC Infectious Diseases* 2009, **9**.

35. Cecchini D, Castillo S, Vecchio C, Sandoval C, Cabral L, Rodriguez Iantorno P, Cassetti I: **[Primary HIV resistance in Buenos Aires metropolitan area]**. *Medicina (B Aires)* 2015, **75**(3):163-168.

36. Chaillon A, Nakazawa M, Wertheim JO, Little SJ, Smith DM, Mehta SR, Gianella S: **No Substantial Evidence for Sexual Transmission of Minority HIV Drug Resistance Mutations in Men Who Have Sex with Men**. *J Virol* 2017, **91**(21).

37. Chamberland AD, S.; Sylla, M.;, Anagounou, S.; Geraldo, N.; Zannou, DM.; Labbé, AC.; Worobey, M.; Alary, M.; Tremblay C.: **Transmission of HIV-1 drug resistance in Benin could jeopardise future treatment options**. *Sexually Transmitted Infections* 2012, **88**(3):179-183.

38. Chan PA, Tashima K, Cartwright CP, Gillani FS, Mintz O, Zeller K, Kantor R: **Short communication: Transmitted drug resistance and molecular epidemiology in antiretroviral naive HIV type 1-infected patients in Rhode Island**. *AIDS Res Hum Retroviruses* 2011, **27**(3):275-281.

39. Chang SY, Chen MY, Lee CN, Sun HY, Ko W, Chang SF, Chang KL, Hsieh SM, Sheng WH, Liu WC *et al*: **Trends of antiretroviral drug resistance in treatment-naive patients with human immunodeficiency virus type 1 infection in Taiwan**. *J Antimicrob Chemother* 2008, **61**(3):689-693.

40. Chang SY, Lin PH, Cheng CL, Chen MY, Sun HY, Hsieh SM, Sheng WH, Su YC, Su LH, Chang SF *et al*: **Prevalence of Integrase Strand Transfer Inhibitors (INSTI) Resistance Mutations in Taiwan**. *Sci Rep* 2016, **6**:35779.

41. Chen I, Connor MB, Clarke W, Marzinke MA, Cummings V, Breaud A, Fogel JM, Laeyendecker O, Fields SD, Donnell D *et al*: **Antiretroviral Drug Use and HIV Drug Resistance Among HIV-Infected Black Men Who Have Sex With Men: HIV Prevention Trials Network 061**. *J Acquir Immune Defic Syndr* 2015, **69**(4):446-452.

42. Chen I, Zhang Y, Cummings V, Cloherty GA, Connor M, Beauchamp G, Griffith S, Rose S, Gallant J, Scott HM *et al*: **Analysis of HIV Integrase Resistance in Black Men Who Have Sex with Men in the United States**. *AIDS Res Hum Retroviruses* 2017, **33**(7):745-748.

43. Chen M, Jia MH, Ma YL, Luo HB, Chen HC, Yang CJ, Dai J, Yang L, Dong LJ, Lu R *et al*: **The changing HIV-1 genetic characteristics and transmitted drug resistance among recently infected population in Yunnan, China**. *Epidemiol Infect* 2018, **146**(6):775-781.

44. Chen M, Ma Y, Chen H, Dai J, Dong L, Yang C, Li Y, Luo H, Zhang R, Jin X *et al*: **HIV-1 genetic transmission networks among men who have sex with men in Kunming, China**. *PLoS One* 2018, **13**(4):e0196548.

45. Chen M, Ma Y, Duan S, Xing H, Yao S, Su Y, Luo H, Yang L, Chen H, Fu L *et al*: **Genetic diversity and drug resistance among newly diagnosed and antiretroviral treatment-naive HIV-infected individuals in western Yunnan: a hot area of viral recombination in China**. *BMC Infect Dis* 2012, **12**:382.

46. Chen MM, Y.; Su, Y.; Yang, L.; Zhang, R.; Yang, C.; Chen, H.; Yan, W.; Shi, Y.; Dong, L.; Chen, L.: **HIV-1 genetic characteristics and transmitted drug resistance among men who have sex with men in Kunming, China**. *PLoS ONE* 2014, **9**(1):no pagination.

47. Chen S, Cai W, He J, Vidal N, Lai C, Guo W, He H, Chen X, Fu L, Peeters M *et al*: **Molecular epidemiology of human immunodeficiency virus type 1 in Guangdong province of southern China**. *PLoS One* 2012, **7**(11):e48747.

48. Cheng CY, Tsai MS, Yang CJ, Cheng SH, Sun HY, Chang SF, Su LH, Su YC, Hung CC, Chang SY: **Patterns of emergent resistance-associated mutations after initiation of non-nucleoside reverse-transcriptase inhibitor-containing antiretroviral regimens in Taiwan: a multicenter cohort study**. *Infect Drug Resist* 2018, **11**:849-859.

49. Chin BS, Choi J, Nam JG, Kee MK, Suh SD, Choi JY, Chu C, Kim SS: **Inverse relationship between viral load and genotypic resistance mutations in Korean patients with primary HIV type 1 infections**. *AIDS Res Hum Retroviruses* 2006, **22**(11):1142-1147.

50. Choi JY, Kim EJ, Park YK, Lee JS, Kim SS: **National survey for drug-resistant variants in newly diagnosed antiretroviral drug-naive patients with HIV/AIDS in South Korea: 1999-2005**. *J Acquir Immune Defic Syndr* 2008, **49**(3):237-242.

51. Coetzee J, Hunt G, Jaffer M, Otwombe K, Scott L, Bongwe A, Ledwaba J, Molema S, Jewkes R, Gray GE: **HIV-1 viraemia and drug resistance amongst female sex workers in Soweto, South Africa: A cross sectional study**. *PLoS One* 2017, **12**(12):e0188606.

52. Colafigli MC, Torti.; Enrico Maria, Trecarichi.; Laura, Albini.; Andrea, Rosi.; Valeria, Micheli.; Nino, Manca et al.: **Evolution of transmitted HIV-1 drug resistance in HIV-1-infected patients in Italy from 2000 to 2010**. *Clinical Microbiology and Infection* 2012, **18**(8):E299-E304.

53. Colby DJ, Crowell TA, Sirivichayakul S, Pinyakorn S, Kroon E, Benjapornpong K, Intasan J, Trichavaroj R, Tovanabutra S, Robb M *et al*: **Declining trend in transmitted drug resistance detected in a prospective cohort study of acute HIV infection in Bangkok, Thailand**. *J Int AIDS Soc* 2016, **19**(1):20966.

54. Colby DP, N.; Sirivichayakul, S.; Prueksakaew, P.; Saengtawan, P.; Trichavaroj, R.; Crowell, T.; Kim, J.; Ananworanich, J.; Phanuphak, P.: **HIV transmitted drug resistance declined from 2009 to 2014 among acutely infected MSM in Bangkok, Thailand**. *Journal of the International AIDS Society* 2015, **18**:73.

55. Collins-Fairclough AM, Dennis AM, Nelson JA, Weir SS, Figueroa JP: **HIV Drug Resistance Surveillance Among Jamaican Men Who Have Sex with Men Should Be Prioritized for Reducing HIV Transmission**. *AIDS Res Hum Retroviruses* 2015, **31**(8):841-844.

56. Cozzi L, D'Arminio M, C F: **Risk of failure in patients with 215 HIV-1 revertants starting their first thymidine analog-containing highly active antiretroviral therapy**. *AIDS* 2004, **18**(2):227-235.

57. Cozzi-Lepri A, Noguera-Julian M, Di Giallonardo F, Schuurman R, Daumer M, Aitken S, Ceccherini-Silberstein F, D'Arminio Monforte A, Geretti AM, Booth CL *et al*: **Low-frequency drug-resistant HIV-1 and risk of virological failure to first-line NNRTI-based ART: a multicohort European case-control study using centralized ultrasensitive 454 pyrosequencing**. *J Antimicrob Chemother* 2015, **70**(3):930-940.

58. Dai L, Li N, Wei F, Li J, Liu Y, Xia W, Zhang T, Guo C, Wang W, Schwartz SA *et al*: **Transmitted antiretroviral drug resistance in the men who have sex with men HIV patient cohort, Beijing, China, 2008-2011**. *Viral Immunol* 2014, **27**(8):392-397.

59. Dai LM, S.; Sykes, D.; Nair, B.; Schwartz, S.: **Quantitative transmitted drug resistance (TDR) variation in acute/ recently infected men who have sex with men (MSM) Chinese HIV patient cohort**. *Journal of Antivirals and Antiretrovirals* 2014, **6**(1):13-21.

60. Davarpanah MA, Motazedian N, Joulaei H, Aghasadeghi MR, Faramarzi H, Aghah E: **Comparison of antiretroviral drug resistance among treatment-naive and treated HIV-infected individuals in Shiraz, Iran**. *Arch Virol* 2018, **163**(1):99-104.

61. De Gascun CF, Waters A, Regan C, O'Halloran J, Farrell G, Coughlan S, Bergin C, Powderly WG, Hall WW: **Documented prevalence of HIV type 1 antiretroviral transmitted drug resistance in Ireland from 2004 to 2008**. *AIDS Res Hum Retroviruses* 2012, **28**(3):276-281.

62. de Medeiros LB, Lacerda HR, Cavalcanti AM, de Albuquerque Mde F: **Primary resistance of human immunodeficiency virus type 1 in a reference center in Recife, Pernambuco, Brazil**. *Mem Inst Oswaldo Cruz* 2006, **101**(8):845-849.

63. de Mendoza C, Rodriguez C, Corral A, del Romero J, Gallego O, Soriano V: **Evidence for differences in the sexual transmission efficiency of HIV strains with distinct drug resistance genotypes**. *Clin Infect Dis* 2004, **39**(8):1231-1238.

64. de Mendoza C, Rodriguez C, Eiros JM, Colomina J, Garcia F, Leiva P, Torre-Cisneros J, Aguero J, Pedreira J, Viciana I *et al*: **Antiretroviral recommendations may influence the rate of transmission of drug-resistant HIV type 1**. *Clin Infect Dis* 2005, **41**(2):227-232.

65. Dean J, Ta Thi TH, Dunford L, Carr MJ, Nguyen LT, Coughlan S, Connell J, Nguyen HT, Hall WW, Nguyen Thi LA: **Prevalence of HIV type 1 antiretroviral drug resistance mutations in Vietnam: a multicenter study**. *AIDS Res Hum Retroviruses* 2011, **27**(7):797-801.

66. Delaugerre C, Rodriguez C, Capitant C, Nere ML, Mercier-Darty M, Carette D, Pialoux G, Cotte L, Charreau I, Molina JM *et al*: **Drug resistance among patients who acquired HIV infection in a preexposure prophylaxis trial**. *AIDS* 2018, **32**(16):2353-2361.

67. Descamps DC, ML.; Montes, B.; Pakianather, S.; Charpentier, C.; Storto, A.; Barin, F.; Dos Santos, G.; Krivine, A.; Delaugerre, C.; Izopet, J.: **Increasing prevalence of transmitted drug resistance mutations and non-B subtype circulation in antiretroviral-naive chronically HIV-infected patients from 2001 to 2006/2007 in France**. *Journal of Antimicrobial Chemotherapy* 2010, **65**(12):2620-2627.

68. Do HT, Nguyen DT, Nguyen LAT, Do DH, Le HX, Trinh XMT, Ton HVN, Sawada I, Kitamura N, Le MN *et al*: **An Alarmingly High Proportion of HIV-1 Isolates Carrying Mutations Corresponding to Resistance to Antiretroviral Drugs among HIV-Positive High-Risk Groups in Central Vietnam: a Substudy of the National Sentinel Survey**. *Jpn J Infect Dis* 2017, **70**(6):621-627.

69. Drescher SvW, V.; Yang, WL.; Böni, J.; Yerly, S.; Shah, C.; Aubert, V.; Klimkait, T.; Taffé, P.; Furrer, H.; Battegay, M: **Treatment-naive individuals are the major source of transmitted HIV-1 drug resistance in men who have sex with men in the Swiss HIV cohort study**. *Clinical Infectious Diseases* 2014, **58**(2):285-294.

70. Dvali NP, MM.; Chkhartishvili, N.; Sharvadze, L.; Gochitashvili, N.; Abutidze, A.; Karchava, M.; DeHovitz, JA.; Tsertsvadze, T. : **Characterization of HIV-1 subtypes and drug resistance mutations among individuals infected with HIV in Georgia**. *Journal of Medical Virology* 2012, **84**(7):1002-1008.

71. Easterbrook PJ, Hertogs K, Waters A, Wills B, Gazzard BG, Larder B: **Low prevalence of antiretroviral drug resistance among HIV-1 seroconverters in London, 1984-1991**. *J Infect* 2002, **44**(2):88-91.

72. Escoto-Delgadillo M, Torres-Mendoza BM, Flores-Soto M, Vazquez-Valls E: **HIV Drug Resistance in Antiretroviral-Naive Patients in Mexico After 10 Years: Is There a Difference?** *AIDS Res Hum Retroviruses* 2016, **32**(12):1219-1222.

73. Eyzaguirre LB, KC.; Nadai, Y.; Patterson, TL.; Ramos, R.; Cruz, MF.; Orozovich, P.; Strathdee, SA.; Carr, JK.: **First molecular surveillance report of HIV type 1 in injecting drug users and female sex workers along the U.S.-Mexico border**. *AIDS Research and Human Retroviruses* 2007, **23**(2):331-334.

74. Fabeni L, Alteri C, Di Carlo D, Orchi N, Carioti L, Bertoli A, Gori C, Forbici F, Continenza F, Maffongelli G *et al*: **Dynamics and phylogenetic relationships of HIV-1 transmitted drug resistance according to subtype in Italy over the years 2000-14**. *J Antimicrob Chemother* 2017, **72**(10):2837-2845.

75. Fabeni L, Alteri C, Orchi N, Gori C, Bertoli A, Forbici F, Montella F, Pennica A, De Carli G, Giuliani M *et al*: **Recent Transmission Clustering of HIV-1 C and CRF17_BF Strains Characterized by NNRTI-Related Mutations among Newly Diagnosed Men in Central Italy**. *PLoS One* 2015, **10**(8):e0135325.

76. Ferreira JL, Rodrigues R, Lanca AM, de Almeida VC, Rocha SQ, Ragazzo TG, Estevam DL, Brigido LF: **Transmitted Drug Resistance among People Living with HIV/Aids at Major Cities of Sao Paulo State, Brazil**. *Adv Virol* 2013, **2013**:878237.

77. Fox J, Dustan S, McClure M, Weber J, Fidler S: **Transmitted drug-resistant HIV-1 in primary HIV-1 infection; incidence, evolution and impact on response to antiretroviral therapy**. *HIV Med* 2006, **7**(7):477-483.

78. Fox JH, S.; Kaye, S.; Dustan, S.; McClure, M.; Fidler, S.; Mackie, NE.: **Prevalence of primary genotypic resistance in a UK centre: Comparison of primary HIV-1 and newly diagnosed treatment-naive individuals**. *AIDS* 2007, **21**(2):237-239.

79. Franca D, Del-Rios NHA, Carneiro M, Guimaraes RA, Caetano KAA, Reis M, Martins RMB, Motta-Castro ARC, Stefani MMA, Teles SA: **HIV-1 infection among crack cocaine users in a region far from the epicenter of the HIV epidemic in Brazil: Prevalence and molecular characteristics**. *PLoS One* 2018, **13**(7):e0199606.

80. Franzetti M, Lai A, Simonetti FR, Bozzi G, De Luca A, Micheli V, Meraviglia P, Corsi P, Bagnarelli P, Almi P *et al*: **High burden of transmitted HIV-1 drug resistance in Italian patients carrying F1 subtype**. *J Antimicrob Chemother* 2012, **67**(5):1250-1253.

81. Garcia G, Saiz De La H, Sanchez P, Garcia B, Ruiz R: **Prevalence of HIV-1 drug resistance mutations among Spanish prison inmates**. *European Journal of Clinical Microbiology and Infectious Diseases* 2006, **25**(11):695-701.

82. Garcia-Morales C, Tapia-Trejo D, Quiroz-Morales VS, Navarro-Alvarez S, Barrera-Arellano CA, Casillas-Rodriguez J, Romero-Mora KA, Gomez-Palacio-Schjetnan M, Murakami-Ogasawara A, Avila-Rios S *et al*: **HIV pretreatment drug resistance trends in three geographic areas of Mexico**. *J Antimicrob Chemother* 2017, **72**(11):3149-3158.

83. Gashnikova NM, Astakhova EM, Gashnikova MP, Bocharov EF, Petrova SV, Pun'ko OA, Popkov AV, Totmenin AV: **HIV-1 Epidemiology, Genetic Diversity, and Primary Drug Resistance in the Tyumen Oblast, Russia**. *BioMed research international* 2016, **2016**:2496280.

84. Ghafari S, Memarnejadian A, Samarbaf-Zadeh A, Mostafavi E, Makvandi M, Salmanzadeh S, Ghadiri A, Jordan MR, Mousavi E, Jahanbakhsh F *et al*: **Prevalence of HIV-1 transmitted drug resistance in recently infected, treatment-naive persons in the Southwest of Iran, 2014-2015**. *Arch Virol* 2017, **162**(9):2737-2745.

85. Gianella S, Morris SR, Anderson C, Spina CA, Vargas MV, Young JA, Richman DD, Little SJ, Smith DM: **Herpes viruses and HIV-1 drug resistance mutations influence the virologic and immunologic milieu of the male genital tract**. *AIDS* 2013, **27**(1):39-47.

86. Gittens MV, Roth WW, Roach T, Stringer HG, Jr., Pieniazek D, Bond VC, Levett PN: **The molecular epidemiology and drug resistance determination of HIV type 1 subtype B infection in Barbados**. *AIDS Res Hum Retroviruses* 2003, **19**(4):313-319.

87. Giuliani M, Montieri S, Palamara G, Latini A, Alteri C, Perno CF, Santoro MM, Rezza G, Ciccozzi M: **Non-B HIV type 1 subtypes among men who have sex with men in Rome, Italy**. *AIDS Res Hum Retroviruses* 2009, **25**(2):157-164.

88. Gong X, Liu Y, Liu FL, Jin L, Wang H, Zheng YT: **A SDF1 genetic variant confers resistance to HIV-1 infection in intravenous drug users in China**. *Infect Genet Evol* 2015, **34**:137-142.

89. González D, C M, Del A: **Clinical, virological and phylogenetic characterization of a multiresistant HIV-1 strain outbreak in naive patients in southern Spain**. *Journal of Antimicrobial Chemotherapy* 2016, **71**(2):357-361.

90. Gonzalez-Domenech CM, Viciana I, Delaye L, Mayorga ML, Palacios R, de la Torre J, Jarilla F, Castano M, Del Arco A, Clavijo E *et al*: **Emergence as an outbreak of the HIV-1 CRF19_cpx variant in treatment-naive patients in southern Spain**. *PLoS One* 2018, **13**(1):e0190544.

91. Grgic I, Lepej SZ, Lunar MM, Poljak M, Vince A, Vrakela IB, Planinic A, Seme K, Begovac J: **The prevalence of transmitted drug resistance in newly diagnosed HIV-infected individuals in Croatia: the role of transmission clusters of men who have sex with men carrying the T215S surveillance drug resistance mutation**. *AIDS Res Hum Retroviruses* 2013, **29**(2):329-336.

92. Guimaraes ML, Marques BC, Bertoni N, Teixeira SL, Morgado MG, Bastos FI, Brazilian Multicity Study Group on Drug M: **Assessing the HIV-1 Epidemic in Brazilian Drug Users: A Molecular Epidemiology Approach**. *PLoS One* 2015, **10**(11):e0141372.

93. Guo J, Yan Y, Zhang J, Ji J, Ge Z, Ge R, Zhang X, Wang H, Chen Z, Luo J: **Genetic characterization and antiretroviral resistance mutations among treatment-naive HIV-infected individuals in Jiaxing, China**. *Oncotarget* 2017, **8**(11):18271-18279.

94. Hanna GJ, Balaguera HU, Freedberg KA, Werner BG, Steger Craven KA, Craven DE, D'Aquila RT: **Drug-selected resistance mutations and non-B subtypes in antiretroviral-naive adults with established human immunodeficiency virus infection**. *J Infect Dis* 2003, **188**(7):986-991.

95. Hightow W, L B, C B, Ii G, T P, Enriquez B: **Transmitted HIV-1 drug resistance among young men of color who have sex with men: A multicenter cohort analysis**. *Journal of Adolescent Health* 2011, **48**(1):94-99.

96. Hiransuthikul A, Wongkanya R, Sirivichayakul S, Trachunthong D, Sungsing T, Pankam T, Phanuphak P, Phanuphak N: **Short Communication: Discordance in Drug Resistance Mutations Between Blood Plasma and Semen or Rectal Secretions Among Newly Diagnosed HIV-1-Infected Thai Men Who Have Sex with Men**. *AIDS Res Hum Retroviruses* 2018, **34**(7):626-628.

97. Horban AS, JJ.; Bakowska, E.; Tobolewska, EJ.; Przybylska-Stengiel, KJ.; Stanczak, GP.; Burkacka, E. : **High prevalence of genotypic resistance to nucleoside reverse transcriptase inhibitors among therapy-naive individuals from the Warsaw cohort**. *Infection* 2002, **30**(6):356-359.

98. Hou LJ, Wang HW, Duan SP, Zhuo Y, Zhou YC, Wu HJ, Shen BS: **The prevalence and determinants of drug-resistance-associated mutations in the HIV-1-infected MSM population of Henan Province in China**. *Arch Virol* 2015, **160**(8):2051-2061.

99. Hsu LY, Subramaniam R, Bacheler L, Paton NI: **Characterization of mutations in CRF01_AE virus isolates from antiretroviral treatment-naive and -experienced patients in Singapore**. *J Acquir Immune Defic Syndr* 2005, **38**(1):5-13.

100. Hua J, Lin H, Ding Y, Qiu D, Wong F, He N: **HIV drug resistance in newly diagnosed adults in a rural prefecture of eastern China**. *Epidemiol Infect* 2015, **143**(3):663-672.

101. Hurt CB, McCoy SI, Kuruc J, Nelson JA, Kerkau M, Fiscus S, McGee K, Sebastian J, Leone P, Pilcher C *et al*: **Transmitted antiretroviral drug resistance among acute and recent HIV infections in North Carolina from 1998 to 2007**. *Antivir Ther* 2009, **14**(5):673-678.

102. Iarikov DE, Irizarry-Acosta M, Martorell C, Hoffman RP, Skiest DJ: **Low prevalence of primary HIV resistance in western Massachusetts**. *J Int Assoc Physicians AIDS Care (Chic)* 2010, **9**(4):227-231.

103. Ibe S, Hotta N, Takeo U, Tawada Y, Mamiya N, Yamanaka K, Utsumi M, Kaneda T: **Prevalence of drug-resistant human immunodeficiency virus type 1 in therapy-naive patients and usefulness of genotype testing**. *Microbiol Immunol* 2003, **47**(7):499-505.

104. Indriati DW, Kotaki T, Khairunisa SQ, Witaningrum AM, Matondang MQY, Ueda S, Nasronudin, Purnama A, Kurniawan D, Kameoka M: **Appearance of Drug Resistance Mutations Among the Dominant HIV-1 Subtype, CRF01_AE in Maumere, Indonesia**. *Curr HIV Res* 2018, **16**(2):158-166.

105. Iqbal HS, Solomon SS, Madhavan V, Solomon S, Balakrishnan P: **Primary HIV-1 drug resistance and polymorphic patterns among injecting drug users (IDUs) in Chennai, Southern India**. *J Int Assoc Physicians AIDS Care (Chic)* 2009, **8**(5):323-327.

106. Jayaraman GC, Archibald CP, Kim J, Rekart ML, Singh AE, Harmen S, Wood M, Sandstrom P: **A population-based approach to determine the prevalence of transmitted drug-resistant HIV among recent versus established HIV infections: results from the Canadian HIV strain and drug resistance surveillance program**. *J Acquir Immune Defic Syndr* 2006, **42**(1):86-90.

107. Jeanette M, Michael J, Lynn E, An T, David A: **Association Between Risk Behaviors and Antiretroviral Resistance in HIV-lnfected Patients Receiving Opioid Agonist Treatment**. *Journal of Addiction Medicine* 2013, **7**(2):102-107.

108. Jeong W, Jung IY, Choi H, Kim JH, Seong H, Ahn JY, Jeong SJ, Ku NS, Kim JM, Choi JY: **Integrase Strand Transfer Inhibitor Resistance Mutations in Antiretroviral Therapy-Naive and Treatment-Experienced HIV Patients in South Korea**. *AIDS Res Hum Retroviruses* 2019, **35**(2):213-216.

109. Jeulin H, Foissac M, Boyer L, Agrinier N, Perrier P, Kennel A, Velay A, Goehringer F, Henard S, Rabaud C *et al*: **Real-life rilpivirine resistance and potential emergence of an E138A-positive HIV strain in north-eastern France**. *J Antimicrob Chemother* 2014, **69**(11):3095-3102.

110. Jiamsakul A, Sirivichayakul S, Ditangco R, Wong KH, Li PC, Praparattanapan J, Phanuphak P, Segubre-Mercado E, Yam WC, Sirisanthana T *et al*: **Transmitted drug resistance in recently infected HIV-positive Individuals from four urban locations across Asia (2007-2010) - TASER-S**. *AIDS Res Ther* 2015, **12**:3.

111. Jiamsakul A, Sungkanuparph S, Law M, Kantor R, Praparattanapan J, Li PC, Phanuphak P, Merati T, Ratanasuwan W, Lee CK *et al*: **HIV multi-drug resistance at first-line antiretroviral failure and subsequent virological response in Asia**. *J Int AIDS Soc* 2014, **17**:19053.

112. Kanizsai S, Ghidan A, Ujhelyi E, Banhegyi D, Nagy K: **Monitoring of drug resistance in therapy-naive HIV infected patients and detection of African HIV subtypes in Hungary**. *Acta Microbiol Immunol Hung* 2010, **57**(1):55-68.

113. Karade S, Patil AA, Ghate M, Kulkarni SS, Kurle SN, Risbud AR, Rewari BB, Gangakhedkar RR: **Short Communication: Limited HIV Pretreatment Drug Resistance Among Adults Attending Free Antiretroviral Therapy Clinic of Pune, India**. *AIDS Res Hum Retroviruses* 2016, **32**(4):377-380.

114. Karlsson A, Bjorkman P, Bratt G, Ekvall H, Gisslen M, Sonnerborg A, Mild M, Albert J: **Low prevalence of transmitted drug resistance in patients newly diagnosed with HIV-1 infection in Sweden 2003-2010**. *PLoS One* 2012, **7**(3):e33484.

115. Keita A, Sereme Y, Pillet S, Coulibaly S, Diallo F, Pozzetto B, Thiero TA, Bourlet T: **Impact of HIV-1 primary drug resistance on the efficacy of a first-line antiretroviral regimen in the blood of newly diagnosed individuals in Bamako, Mali**. *J Antimicrob Chemother* 2019, **74**(1):165-171.

116. Kostrikis LG, Hezka J, Stylianou DC, Kostaki E, Andreou M, Kousiappa I, Paraskevis D, Demetriades I: **HIV-1 transmission networks across Cyprus (2010-2012)**. *PLoS One* 2018, **13**(4):e0195660.

117. Kotaki TK, SQ.; Witaningrum, AM.; Sukartiningrum, SD.; Diansyah, MN.; Rahayu, RP.; Kameoka, M.: **HIV-1 transmitted drug resistance mutations among antiretroviral therapy-Naive individuals in Surabaya, Indonesia**. *AIDS Research and Therapy* 2015, **12**(1):no pagination.

118. Kroon E, Pham PT, Sirivichayakul S, Trichavaroj R, Colby DJ, Pinyakorn S, Phanuphak N, Sanders-Buell E, van Griensven F, Kijak GH *et al*: **Transmission dynamics among participants initiating antiretroviral therapy upon diagnosis of early acute HIV-1 infection in Thailand**. *AIDS* 2018, **32**(16):2373-2381.

119. Lai A, Violin M, Ebranati E, Franzetti M, Micheli V, Gismondo MR, Capetti A, Meraviglia P, Simonetti FR, Bozzi G *et al*: **Transmission of resistant HIV type 1 variants and epidemiological chains in Italian newly diagnosed individuals**. *AIDS Res Hum Retroviruses* 2012, **28**(8):857-865.

120. Lan YC, Elbeik T, Dileanis J, Ng V, Chen YJ, Leu HS, Cheng SH, Wong JC, Wong WW, Chen YM: **Molecular epidemiology of HIV-1 subtypes and drug resistant strains in Taiwan**. *J Med Virol* 2008, **80**(2):183-191.

121. Lapadula G, Izzo I, Gargiulo F, Paraninfo G, Castelnuovo F, Quiros-Roldan E, Cologni G, Ceresoli F, Manca N, Carosi G *et al*: **Updated prevalence of genotypic resistance among HIV-1 positive patients naive to antiretroviral therapy: a single center analysis**. *J Med Virol* 2008, **80**(5):747-753.

122. Leszczyszyn P: **Differences in the integrase and reverse transcriptase transmitted resistance patterns in Northern Poland**. *Infection, Genetics and Evolution* 2017, **49**:122-129.

123. Leszczyszyn P, Witak J, Bociaga J, Mozer L: **Transmitted HIV drug resistance in antiretroviral-treatment-naive patients from Poland differs by transmission category and subtype**. *Journal of Antimicrobial Chemotherapy* 2015, **70**(1):233-242.

124. Li H, Chang S, Han Y, Zhuang D, Li L, Liu Y, Liu S, Bao Z, Zhang W, Song H *et al*: **The prevalence of drug resistance among treatment-naive HIV-1-infected individuals in China during pre- and post- 2004**. *BMC Infect Dis* 2016, **16**(1):605.

125. Li JF, Linley L, Kline R, Ziebell R, Heneine W, Johnson JA: **Sensitive sentinel mutation screening reveals differential underestimation of transmitted HIV drug resistance among demographic groups**. *AIDS* 2016, **30**(9):1439-1445.

126. Li L, Wei D, Hsu WL, Li T, Gui T, Wood C, Liu Y, Li H, Bao Z, Liu S *et al*: **CRF07_BC Strain Dominates the HIV-1 Epidemic in Injection Drug Users in Liangshan Prefecture of Sichuan, China**. *AIDS Res Hum Retroviruses* 2015, **31**(5):479-487.

127. Li LH, N.; Lu, J.; Li, T.; Zhong, X.; Wu, H.; Rayner, S.; Chen, L.; Liu, Y.; Wang, X.; Li, H.; Li, J: **Genetic characterization and transmitted drug resistance of the HIV type 1 epidemic in men who have sex with men in Beijing, China**. *AIDS Research and Human Retroviruses* 2013, **29**(3):633-637.

128. Li LS, G.; Liang, S.; Li, J.; Li, T,; Wang, Z.; Liu, W.; Yang, S.; Liu, Y.; Wang, X,; Li, J.: **Different Distribution of HIV-1 Subtype and Drug Resistance Were Found among Treatment Naïve Individuals in Henan, Guangxi, and Yunnan Province of China**. *PLoS ONE* 2013, **8**(10).

129. Li XX, Y.; Cheng, H.; Lin, Y.; Zhou, L.; Ning, Z.; Wang, X.; Yu, X.; Zhang, W.; Shen, F.; Zheng, X.; Gai, J.; Li, X.; Kang, L.; Nyambi, P.; Wang, Y.; Zhuang, M.; Pan, Q.; Zhuang, X.; Zhong, P.: **HIV-1 genetic diversity and its impact on baseline CD4+T cells and viral loads among recently infected men who have sex with men in Shanghai, China**. *PLoS ONE* 2015, **10**(6):no pagination.

130. Li Y, Gu L, Han Y, Xie J, Wang H, Lv W, Song X, Li Y, Iwamoto A, Ishida T *et al*: **HIV-1 subtype B/B' and baseline drug resistance mutation are associated with virologic failure: a multicenter cohort study in China**. *J Acquir Immune Defic Syndr* 2015, **68**(3):289-297.

131. Liao L XH, Shang H, Li J, Zhong P, Kang L, Cheng H, Si X, Jiang S, Li X, Shao Y: **The prevalence of transmitted antiretroviral drug resistance in treatment-naive HIV-infected individuals in China**. *Journal of Acquired Immune Deficiency Syndromes* 2010, **53**:S10-14.

132. Lima YA, Reis MN, Cardoso LP, Stefani MM: **HIV-1 infection and pregnancy in young women in Brazil: socioeconomic and drug resistance profiles in a cross-sectional study**. *BMJ Open* 2016, **6**(7):e010837.

133. Lindström AO, A; Huigen, M; Nijhuis, M; Berglund, T; Bratt, G; Sandström, E; Albert, J: **HIV-1 transmission cluster with M41L 'singleton' mutation and decreased transmission of resistance in newly diagnosed Swedish homosexual men**. *Antiviral Therapy* 2006, **11**(8):1031-1039.

134. Lopes Maia T, Inácio B, M A, Lindenmeyer G, Gonçalves M: **Trends in drug resistance mutations in antiretroviral-naïve intravenous drug users of Rio de Janeiro**. *Journal of Medical Virology* 2006, **78**(6):764-769.

135. López D, M Á, Gómez C, J M, J M: **Trends in transmission of drug resistance and prevalence of Non-B subtypes in patients with acute or recent HIV-1 infection in Barcelona in the last 16 years (1997-2012)**. *PLoS ONE* 2015, **10**(6).

136. Lu X, Chen S, Zhao H, Li Y, Wang Y, Zhang Y, Lian K, Zhao C, Cui Z: **Baseline Investigation of HIV-1 Primary Drug Resistance Among Newly Diagnosed Treatment-Naive HIV-1 Individuals in Hebei, China**. *AIDS Res Hum Retroviruses* 2018, **34**(12):1083-1089.

137. Lu X, Kang X, Liu Y, Li Y, Chen S, Li J, Cui Z: **Surveillance of Transmitted Drug Resistance in HIV-1-Infected Youths Aged 16 to 25 Years, a Decade After Scale-up of Antiretroviral Therapy in Hebei, China**. *AIDS Res Hum Retroviruses* 2017, **33**(4):359-363.

138. Lunar MM, Zidovec Lepej S, Tomazic J, Vovko TD, Pecavar B, Turel G, Maver M, Poljak M: **HIV-1 transmitted drug resistance in Slovenia and its impact on predicted treatment effectiveness: 2011-2016 update**. *PLoS One* 2018, **13**(4):e0196670.

139. Lunar MŽL, S.; Abecasis, AB.; Tomažič, J.; Vidmar, L.; Karner, P.; Vovko, TD.; Pečavar, B.; Maver, PJ.; Seme, K.; & Poljak, M. : **Short communication: Prevalence of HIV type 1 transmitted drug resistance in Slovenia: 2005-2010**. *AIDS Research and Human Retroviruses* 2013, **29**(2):343-349.

140. Machado DM, Delwart EL, Diaz RS, de Oliveira CF, Alves K, Rawal BD, Sullivan M, Gwinn M, Clark KA, Busch MP: **Use of the sensitive/less-sensitive (detuned) EIA strategy for targeting genetic analysis of HIV-1 to recently infected blood donors**. *AIDS* 2002, **16**(1):113-119.

141. Machado L: **Transmitted HIV type-1 drug resistance in newly diagnosed patients from Havana City: 2009-2012**. *Journal of the International AIDS Society* 2013, **16**:21-22.

142. Maia Teixeira SL, Bastos FI, Hacker MA, Guimaraes ML, Morgado MG: **Trends in drug resistance mutations in antiretroviral-naive intravenous drug users of Rio de Janeiro**. *J Med Virol* 2006, **78**(6):764-769.

143. Maljkovic I, Wilbe K, Solver E, Alaeus A, Leitner T: **Limited transmission of drug-resistant HIV type 1 in 100 Swedish newly detected and drug-naive patients infected with subtypes A, B, C, D, G, U, and CRF01_AE**. *AIDS Res Hum Retroviruses* 2003, **19**(11):989-997.

144. Masquelier B, Bhaskaran K, Pillay D, Gifford R, Balestre E, Jorgensen LB, Pedersen C, van der Hoek L, Prins M, Balotta C *et al*: **Prevalence of transmitted HIV-1 drug resistance and the role of resistance algorithms: data from seroconverters in the CASCADE collaboration from 1987 to 2003**. *J Acquir Immune Defic Syndr* 2005, **40**(5):505-511.

145. McFaul KM, Lim C, Jones R, Asboe D, Pozniak A, Sonecha S, Nwokolo N, Boffito M: **Transmitted antiretroviral resistance in a large HIV directorate 2011-2014: a response**. *AIDS* 2015, **29**(7):861-862.

146. Memarnejadian AM, S.; Mansouri, SA.; Sadeghi, L.; Vahabpour, R.; Aghasadeghi, MR.; Mostafavi, E.; Abdi, M. : **Transmitted drug resistance mutations in antiretroviral-naïve injection drug users with chronic HIV-1 infection in Iran**. *PLoS ONE* 2015, **10**(5).

147. Mendoza Y, Castillo Mewa J, Martinez AA, Zaldivar Y, Sosa N, Arteaga G, Armien B, Bautista CT, Garcia-Morales C, Tapia-Trejo D *et al*: **HIV-1 Antiretroviral Drug Resistance Mutations in Treatment Naive and Experienced Panamanian Subjects: Impact on National Use of EFV-Based Schemes**. *PLoS One* 2016, **11**(4):e0154317.

148. Menezes P, Rosen D, Wohl DA, Kiziah N, Sebastian J, Eron JJ, Jr., White B: **Low prevalence of antiretroviral resistance among HIV type 1-positive prisoners in the Southeast United States**. *AIDS Res Hum Retroviruses* 2013, **29**(1):136-141.

149. Metzner KJ, Rauch P, von Wyl V, Leemann C, Grube C, Kuster H, Boni J, Weber R, Gunthard HF: **Efficient suppression of minority drug-resistant HIV type 1 (HIV-1) variants present at primary HIV-1 infection by ritonavir-boosted protease inhibitor-containing antiretroviral therapy**. *J Infect Dis* 2010, **201**(7):1063-1071.

150. Mezei M, Ay E, Koroknai A, Toth R, Balazs A, Bakos A, Gyori Z, Banati F, Marschalko M, Karpati S *et al*: **Molecular epidemiological analysis of env and pol sequences in newly diagnosed HIV type 1-infected, untreated patients in Hungary**. *AIDS Res Hum Retroviruses* 2011, **27**(11):1243-1247.

151. Moura ME, da Guarda Reis MN, Lima YA, Eulalio KD, Cardoso LP, Stefani MM: **HIV-1 transmitted drug resistance and genetic diversity among patients from Piaui State, Northeast Brazil**. *J Med Virol* 2015, **87**(5):798-806.

152. Moura ME, Reis MN, Lima YA, Eulalio KD, Cardoso LP, Stefani MM: **Low rate of transmitted drug resistance may indicate low access to antiretroviral treatment in Maranhao State, northeast Brazil**. *AIDS Res Hum Retroviruses* 2015, **31**(2):250-254.

153. Oette M, Reuter S, Kaiser R, Lengauer T, Fatkenheuer G, Knechten H, Hower M, Pfister H, Haussinger D, group RS: **Epidemiology of transmitted drug resistance in chronically HIV-infected patients in Germany: the RESINA study 2001-2009**. *Intervirology* 2012, **55**(2):154-159.

154. Olson A, Bannert N, Sonnerborg A, de Mendoza C, Price M, Zangerle R, Chaix ML, Prins M, Kran AB, Gill J *et al*: **Temporal trends of transmitted HIV drug resistance in a multinational seroconversion cohort**. *AIDS* 2018, **32**(2):161-169.

155. Osman S, Lihana RW, Kibaya RM, Ishizaki A, Bi X, Okoth FA, Ichimura H, Lwembe RM: **Diversity of HIV type 1 and drug resistance mutations among injecting drug users in Kenya**. *AIDS Res Hum Retroviruses* 2013, **29**(1):187-190.

156. Palma AA, F.; Duque, V.; Borges, F.; Paixão, MT.; Camacho, R.; : **Molecular epidemiology and prevalence of drug resistance-associated mutations in newly diagnosed HIV-1 patients in Portugal**. *Infection, Genetics and Evolution* 2007, **7**(3):391-398.

157. Pando MA, Eyzaguirre LM, Carrion G, Montano SM, Sanchez JL, Carr JK, Avila MM: **High genetic variability of HIV-1 in female sex workers from Argentina**. *Retrovirology* 2007, **4**:58.

158. Pando MG-C, M.; Vignoles, M.; Rubio, AE.; dos Ramos Farias, MS.; Vila, M.; Rossi, D.; Ralón, G.; Marone, R.; Reynaga, E.; Sosa, J. : **Incidence of HIV type 1 infection, antiretroviral drug resistance, and molecular characterization in newly diagnosed individuals in Argentina: A global fund project**. *AIDS Research and Human Retroviruses* 2011, **27**(1):17-23.

159. Paraskevis D, Kostaki E, Gargalianos P, Xylomenos G, Lazanas M, Chini M, Skoutelis A, Papastamopoulos V, Paraskeva D, Antoniadou A *et al*: **Transmission Dynamics of HIV-1 Drug Resistance among Treatment-Naive Individuals in Greece: The Added Value of Molecular Epidemiology to Public Health**. *Genes (Basel)* 2017, **8**(11).

160. Paraskevis D, Magiorkinis E, Katsoulidou A, Hatzitheodorou E, Antoniadou A, Papadopoulos A, Poulakou G, Paparizos V, Botsi C, Stavrianeas N *et al*: **Prevalence of resistance-associated mutations in newly diagnosed HIV-1 patients in Greece**. *Virus Res* 2005, **112**(1-2):115-122.

161. Payne BA, Nsutebu EF, Hunter ER, Olarinde O, Collini P, Dunbar JA, Basta MS, Elston JW, Schmid ML, Thaker H *et al*: **Low prevalence of transmitted antiretroviral drug resistance in a large UK HIV-1 cohort**. *J Antimicrob Chemother* 2008, **62**(3):464-468.

162. Paz B, B S, I. L D: **Transmitted drug resistance and type of infection in newly diagnosed HIV-1 individuals in Honduras**. *Journal of Clinical Virology* 2010, **49**(4):239-244.

163. Pernas B, Mena A, Canizares A, Grandal M, Castro-Iglesias A, Pertega S, Pedreira JD, Poveda E: **Trends on epidemiological, virological, and clinical features among newly diagnosed HIV-1 persons in Northwest Spain over the last 10 years**. *J Med Virol* 2015, **87**(8):1319-1326.

164. Petroni A, Deluchi G, Pryluka D, Rotryng F, Bortolozzi R, Lopardo G, Bouzas MB, Zapiola I, Garone D, Rodriguez C *et al*: **Update on primary HIV-1 resistance in Argentina: emergence of mutations conferring high-level resistance to nonnucleoside reverse transcriptase inhibitors in drug-naive patients**. *J Acquir Immune Defic Syndr* 2006, **42**(4):506-510.

165. Pillay D, Bhaskaran K, Jurriaans S, Prins M, Masquelier B, Dabis F, Gifford R, Nielsen C, Pedersen C, Balotta C *et al*: **The impact of transmitted drug resistance on the natural history of HIV infection and response to first-line therapy**. *AIDS* 2006, **20**(1):21-28.

166. Pineda-Pena AC, Schrooten Y, Vinken L, Ferreira F, Li G, Trovao NS, Khouri R, Derdelinckx I, De Munter P, Kucherer C *et al*: **Trends and predictors of transmitted drug resistance (TDR) and clusters with TDR in a local Belgian HIV-1 epidemic**. *PLoS One* 2014, **9**(7):e101738.

167. Pinto A, Carrera A, Salem H, Thapa K, Shaik A, Petoumenos K, Cunningham P, Garsia R, Dwyer D, Cooper DA *et al*: **Evolution of HIV-1 surveillance drug resistance mutations over ten years in New South Wales, Australia**. *AIDS Res Hum Retroviruses* 2017.

168. Pontali E, Ventura A, Bruzzone B, Icardi G, Ferrari F: **Unexpected high rate of wild-type HIV-1 genotype among inmates failing antiretroviral therapy**. *HIV Clin Trials* 2008, **9**(5):341-347.

169. Pouga L, Santoro MM, Charpentier C, Di Carlo D, Romeo I, Artese A, Alcaro S, Antinori A, Wirden M, Perno CF *et al*: **New resistance mutations to nucleoside reverse transcriptase inhibitors at codon 184 of HIV-1 reverse transcriptase (M184L and M184T)**. *Chem Biol Drug Des* 2019, **93**(1):50-59.

170. Prellwitz IM, Alves BM, Ikeda ML, Kuhleis D, Picon PD, Jarczewski CA, Osorio MR, Sanchez A, Seuanez HN, Larouze B *et al*: **HIV behind bars: human immunodeficiency virus cluster analysis and drug resistance in a reference correctional unit from southern Brazil**. *PLoS One* 2013, **8**(7):e69033.

171. Public Health Agency of C: **HIV–1 strain and transmitted drug resistance in Canada: Surveillance report to December 31, 2008. Centre for Communicable Diseases and Infection Control, Public Health Agency of Canada**. 2008.

172. Qi H, Zhao K, Xu F, Zhang X, Zhang Z, Yang L, Li C, Liang X, Guo W, Chen S *et al*: **HIV-1 diversity, drug-resistant mutations, and viral evolution among high-risk individuals in phase II HIV vaccine trial sites in southern China**. *PLoS One* 2013, **8**(7):e68656.

173. Quang D, Nhan T, Yen N, Thuong V, Duc B, Thu Khanh H, Duong D, Van K, Phuc D, Anh Q *et al*: **Pretreatment HIV-1 drug resistance to first-line drugs: results from a baseline assessment of a large cohort initiating ART in Vietnam, 2009-10**. *Journal of Antimicrobial Chemotherapy (JAC)* 2015, **70**(3):941-947.

174. Resistance UKCGoMtToHD: **Analysis of prevalence of HIV-1 drug resistance in primary infections in the United Kingdom**. *BMJ* 2001, **322**(7294):1087-1088.

175. Reuter S, Oette M, Sichtig N, Kaiser R, Balduin M, Jensen B, Haussinger D, Group RS: **Changes in the HIV-1 mutational profile before first-line HAART in the RESINA cohort**. *J Med Virol* 2011, **83**(2):187-195.

176. Ristig MB, Arens MQ, Kennedy M, Powderly W, Tebas P: **Increasing prevalence of resistance mutations in antiretroviral-naive individuals with established HIV-1 infection from 1996-2001 in St. Louis**. *HIV Clin Trials* 2002, **3**(2):155-160.

177. Riva C, Lai A, Caramma I, Corvasce S, Violin M, Deho L, Prati F, Rossi C, Colombo MC, Capetti A *et al*: **Transmitted HIV Type 1 drug resistance and Non-B subtypes prevalence among seroconverters and newly diagnosed patients from 1992 to 2005 in Italy**. *AIDS Res Hum Retroviruses* 2010, **26**(1):41-49.

178. Ross L, Lim ML, Liao Q, Wine B, Rodriguez AE, Weinberg W, Shaefer M: **Prevalence of antiretroviral drug resistance and resistance-associated mutations in antiretroviral therapy-naive HIV-infected individuals from 40 United States cities**. *HIV Clin Trials* 2007, **8**(1):1-8.

179. Rossetti B, Di Giambenedetto S, Torti C, Postorino MC, Punzi G, Saladini F, Gennari W, Borghi V, Monno L, Pignataro AR *et al*: **Evolution of transmitted HIV-1 drug resistance and viral subtypes circulation in Italy from 2006 to 2016**. *HIV Med* 2018, **19**(9):619-628.

180. Roudinskii NI, Sukhanova AL, Kazennova EV, Weber JN, Pokrovsky VV, Mikhailovich VM, Bobkov AF: **Diversity of human immunodeficiency virus type 1 subtype A and CRF03_AB protease in Eastern Europe: selection of the V77I variant and its rapid spread in injecting drug user populations**. *J Virol* 2004, **78**(20):11276-11287.

181. Sagir AO, M.; Kaiser, R.; Däumer, M.; Fätkenheuer, G.; Rockstroh, JK.; Knechten, H.; Schmutz, G.; Hower, M.; Emmelkamp, J.; Pfister. H.; Häussinger, D.; RESINA Study Team.: **Trends of prevalence of primary HIV drug resistance in Germany**. *Journal of Antimicrobial Chemotherapy* 2007, **60**(4):843-848.

182. Sanabani SS, Pastena ER, da Costa AC, Martinez VP, Kleine-Neto W, de Oliveira AC, Sauer MM, Bassichetto KC, Oliveira SM, Tomiyama HT *et al*: **Characterization of partial and near full-length genomes of HIV-1 strains sampled from recently infected individuals in Sao Paulo, Brazil**. *PLoS One* 2011, **6**(10):e25869.

183. Sapozhnikov J, Young JD, Patel M, Chiampas TD, Vaughn P, Badowski ME: **Prevalence of HIV-1 transmitted drug resistance in the incarcerated population**. *HIV Med* 2017, **18**(10):756-763.

184. Shen Y, Su B, Wu J, Qin Y, Jin L, Miao L, Liu A, Cheng X: **The prevalence of transmitted HIV drug resistance among MSM in Anhui province, China**. *AIDS Res Ther* 2014, **11**:19.

185. Skoura L, Metallidis S, Buckton AJ, Mbisa JL, Pilalas D, Papadimitriou E, Papoutsi A, Haidich AB, Chrysanthidis T, Tsachouridou O *et al*: **Molecular and epidemiological characterization of HIV-1 infection networks involving transmitted drug resistance mutations in Northern Greece**. *J Antimicrob Chemother* 2011, **66**(12):2831-2837.

186. Smolen J, Kruszynski P, Bratosiewicz-Wasik J, Witor A, Wasik TJ: **Retrospective analysis of the HIV-1 reverse transcriptase inhibitors' resistance in Silesia, Poland**. *Med Sci Monit* 2011, **17**(2):BR42-47.

187. Smolen-Dzirba J, Rosinska M, Kruszynski P, Bratosiewicz-Wasik J, Wojtyczka R, Janiec J, Szetela B, Beniowski M, Bociaga-Jasik M, Jablonowska E *et al*: **Prevalence of Transmitted Drug-Resistance Mutations and Polymorphisms in HIV-1 Reverse Transcriptase, Protease, and gp41 Sequences Among Recent Seroconverters in Southern Poland**. *Med Sci Monit* 2017, **23**:682-694.

188. Sprinz E, Netto EM, Patelli M, Lima JS, Furtado JJ, da Eira M, Zajdenverg R, Madruga JV, Lewi DS, Machado AA *et al*: **Primary antiretroviral drug resistance among HIV type 1-infected individuals in Brazil**. *AIDS Res Hum Retroviruses* 2009, **25**(9):861-867.

189. Ssemwanga D, Ndembi N, Lyagoba F, Magambo B, Kapaata A, Bukenya J, Lubega GW, Bertagnolio S, Vandepitte J, Grosskurth H *et al*: **Transmitted antiretroviral drug resistance among drug-naive female sex workers with recent infection in Kampala, Uganda**. *Clin Infect Dis* 2012, **54 Suppl 4**:S339-342.

190. Stecher M, Chaillon A, Eis-Hubinger AM, Lehmann C, Fatkenheuer G, Wasmuth JC, Knops E, Vehreschild JJ, Mehta S, Hoenigl M: **Pretreatment human immunodeficiency virus type 1 (HIV-1) drug resistance in transmission clusters of the Cologne-Bonn region, Germany**. *Clin Microbiol Infect* 2019, **25**(2):253 e251-253 e254.

191. Steegen K, Carmona S, Bronze M, Papathanasopoulos MA, van Zyl G, Goedhals D, MacLeod W, Sanne I, Stevens WS: **Moderate Levels of Pre-Treatment HIV-1 Antiretroviral Drug Resistance Detected in the First South African National Survey**. *PLoS One* 2016, **11**(12):e0166305.

192. Stekler JM, J.; Milne, R.; Tapia, KA.; Mykhalchenko, K.; Holte, S.; Maenza, J.; Stevens, CE.; Buskin, SE.; Mullins, JI.; Frenkel, LM.; Collier, AC. : **Lack of resistance to integrase inhibitors among antiretroviral-naive subjects with primary HIV-1 infection, 2007-2013**. *Antiviral Therapy* 2015, **20**(1):77-80.

193. Stone DR, Corcoran C, Wurcel A, McGovern B, Quirk J, Brewer A, Sutton L, D'Aquila RT: **Antiretroviral drug resistance mutations in antiretroviral-naive prisoners**. *Clin Infect Dis* 2002, **35**(7):883-886.

194. Sullivan AS, P.; Sandre, R.; Harrigan, RP.; Archibald, C.; Halverson, J.; Rank, C.; Brooks, J.; Burchell, A.; Wu, K.; Dowdall-Smith, S.; Latendre-Paquette, J.; Remis, RS.: **Follow-up investigation of a cluster of treatment-naive HIV-infected patients with multi-drug resistance in Sudbury, Ontario**. *Canadian Journal of Infectious Diseases and Medical Microbiology* 2013, **24**:38A.

195. Sullivan PS, Buskin SE, Turner JH, Cheingsong R, Saekhou A, Kalish ML, Jones JL, Respess R, Kovacs A, Heneine W: **Low prevalence of antiretroviral resistance among persons recently infected with human immunodeficiency virus in two US cities**. *Int J STD AIDS* 2002, **13**(8):554-558.

196. Tamalet C, Tissot-Dupont H, Motte A, Tourres C, Dhiver C, Ravaux I, Poizot-Martin I, Dieng T, Tomei C, Bregigeon S *et al*: **Emergence of uncommon HIV-1 non-B subtypes and circulating recombinant forms and trends in transmission of antiretroviral drug resistance in patients with primary infection during the 2013-2015 period in Marseille, Southeastern France**. *J Med Virol* 2018, **90**(10):1559-1567.

197. Taniguchi T, Nurutdinova D, Grubb JR, Onen NF, Shacham E, Donovan M, Overton ET: **Transmitted drug-resistant HIV type 1 remains prevalent and impacts virologic outcomes despite genotype-guided antiretroviral therapy**. *AIDS Res Hum Retroviruses* 2012, **28**(3):259-264.

198. Tanuma JQ, VM.; Hachiya, A.; Joya, A.; Watanabe, K.; Gatanaga, H.; Chau, NV.; Chinh, NT.; Oka, S.: **Low prevalence of transmitted drug resistance of Hiv-1 during 2008-2012 antiretroviral therapy scaling up in Southern Vietnam**. *Journal of Acquired Immune Deficiency Syndromes* 2014, **66**(4):358-364.

199. Temereanca A, Ene L, Mehta S, Manolescu L, Duiculescu D, Ruta S: **Transmitted HIV drug resistance in treatment-naive Romanian patients**. *J Med Virol* 2013, **85**(7):1139-1147.

200. Todesco E, Charpentier C, Bertine M, Wirden M, Storto A, Desire N, Grude M, Nguyen T, Sayon S, Yazdanpanah Y *et al*: **Disparities in HIV-1 transmitted drug resistance detected by ultradeep sequencing between men who have sex with men and heterosexual populations**. *HIV Med* 2017, **18**(9):696-700.

201. Torian LV, Forgione LA: **Transmitted antiretroviral drug resistance in New York City, 2006-2010: the first five years of routine genotype surveillance**. *J Acquir Immune Defic Syndr* 2013, **63**(3):e119-122.

202. Tossonian HK, Raffa JD, Grebely J, Viljoen M, Mead A, Khara M, McLean M, Krishnamurthy A, DeVlaming S, Conway B: **Primary drug resistance in antiretroviral-naive injection drug users**. *Int J Infect Dis* 2009, **13**(5):577-583.

203. Tostevin A, White E, Dunn D, Croxford S, Delpech V, Williams I, Asboe D, Pozniak A, Churchill D, Geretti AM *et al*: **Recent trends and patterns in HIV-1 transmitted drug resistance in the United Kingdom**. *HIV Med* 2017, **18**(3):204-213.

204. Tran VI, A.; Nguyen, CH.; Hoang, HT.; Pham, HV.; Bi, X.; Van Pham, T.; Ichimura, H.: **No increase of drug-resistant HIV type 1 prevalence among drug-naive individuals in northern vietnam**. *AIDS Research and Human Retroviruses* 2012, **28**(10):1349-1351.

205. Tsai HC, IT.; Wu, KS.; Tseng, YT.; Sy, CL.; Chen, JK.; Lee, SJ.; Chen, YS.: **High prevalence of genotypic resistance to integrase inhibitors of HIV-1 strains in Taiwan**. *Topics in Antiviral Medicine* 2015, **23**:260-261.

206. Tsai HC, Chen IT, Wu KS, Tseng YT, Sy CL, Chen JK, Lee SS, Chen YS: **HIV-1 integrase strand-transfer inhibitor resistance in southern Taiwan**. *Oncotarget* 2018, **9**(38):24927-24935.

207. Tupinambas U, Duani H, Martins AV, Aleixo AW, Greco DB: **Transmitted human immunodeficiency virus-1 drug resistance in a cohort of men who have sex with men in Belo Horizonte, Brazil--1996-2012**. *Mem Inst Oswaldo Cruz* 2013, **108**(4):470-475.

208. Turner DA, S.; Chalom, S.; Penn, O.; Pupko, T.; Katchman, E.; Matus, N.; Tellio, H.; Katzir, M.; Avidor, B.: **Emergence of an HIV-1 cluster harbouring the major protease L90M mutation among treatment-naive patients in Tel Aviv, Israel**. *HIV Medicine* 2012:no pagination.

209. Van D, Van D: **Prevalence and epidemiology of HIV type 1 drug resistance among newly diagnosed therapy-naive patients in Belgium from 2003 to 2006**. *AIDS Research and Human Retroviruses* 2008, **24**(3):355-362.

210. van de V, C. A B, van C, van der V: **Virological failure and drug resistance during first line anti-retroviral treatment in Indonesia**. *Journal of Medical Virology* 2013, **85**(8):1394-1401.

211. Van De V, J C: **Patterns of transmitted HIV drug resistance in Europe vary by risk group**. *PLoS ONE* 2014, **9**(4).

212. Weinstock HZ, I.; Heneine, W.; Bennett, D.; Garcia-Lerma, JG.; Douglas, JM Jr.; LaLota, M.; Dickinson, G.; Schwarcz, S.; Torian, L.; Wendell, D.; Paul, S.; Goza, GA.; Ruiz, J.; Boyett, B.; Kaplan, JE.: **The epidemiology of antiretroviral drag resistance among drug-naive HIV-1-infected persons in 10 US cities**. *Journal of Infectious Diseases* 2004, **189**(12):2174-2180.

213. Weng YW, Tsai HC, Lee SS, Wu KS, Sy CL, Chen JK, Chen YS: **Prevalence and associated factors for HIV-1 transmitted drug resistance in voluntary clients for counseling and testing in Southern Taiwan**. *J Microbiol Immunol Infect* 2016, **49**(4):487-493.

214. Wensing AV, J.; van de Vijver, DA.; Albert, J.; Asjo, B.; Balotta, C.; Camacho, R.; Coughlan, S.; Grossman, Z.; Horban, A.; Kucherer, C.: **Transmission of drug-resistant HIV-1 in Europe remains limited to single classes**. *AIDS* 2008, **22**(5):625-635.

215. Wheeler WZ, RA.; Zabina, H.; Pieniazek, D.; Prejean, J.; Bodnar, UR.; Mahle, KC.; Heneine, W.; Johnson, JA.; Hall, HI. : **Prevalence of transmitted drug resistance associated mutations and HIV-1 subtypes in new HIV-1 diagnoses, U.S.-2006**. *AIDS* 2010, **24**(8):1203-1212.

216. Woodson E, Goldberg A, Michelo C, Basu D, Tao S, Schinazi R, Jiang Y, Kilembe W, Karita E, Allen S *et al*: **HIV transmission in discordant couples in Africa in the context of antiretroviral therapy availability**. *AIDS* 2018, **32**(12):1613-1623.

217. Xu Y, Peng X, Peng X, Ji S, Chen B, Wang L, Lu X, Xie T, Sun T, Wang H *et al*: **Characterization of HIV-1 subtypes and transmitted drug resistance among treatment-naive HIV-infected individuals in Zhejiang, China, 2014-2017**. *Arch Virol* 2018, **163**(8):2233-2237.

218. Yan HD, Y.; Wong, FY.; Ning, Z.; Zheng, T.; Nehl, EJ.; He, N.: **Epidemiological, molecular characteristics**

**of, H. I. V. infection among money boys**

**general men who have sex with men in, Shanghai**. *Infection, Genetics and Evolution* 2015, **31**:135-141.

219. Yan M, Zhao K, Du J, Li L, Wu D, Xu S, Zeng X, Wang G, Yu XF: **HIV-1 diversity and drug-resistant mutations in infected individuals in Changchun, China**. *PLoS One* 2014, **9**(6):e100540.

220. Yang C, Liu S, Zhang T, Hou Y, Liu X, Gao Y, Yang G, Wang Z, Chen H, Li M *et al*: **Transmitted antiretroviral drug resistance and thumb subdomain polymorphisms among newly HIV type 1 diagnosed patients infected with CRF01_AE and CRF07_BC virus in Guangdong Province, China**. *AIDS Res Hum Retroviruses* 2012, **28**(12):1723-1728.

221. Yang JX, H.; Niu, J.; Liao, L.; Ruan, Y.; He, X.; Feng, Y.; Li, Z.; Shao, Y.: **The emergence of HIV-1 primary drug resistance genotypes among treatment-naive men who have sex with men in high-prevalence areas in China**. *Archives of Virology* 2013, **158**(4):839-844.

222. Yang WL, Kouyos R, Scherrer AU, Boni J, Shah C, Yerly S, Klimkait T, Aubert V, Furrer H, Battegay M *et al*: **Assessing the Paradox Between Transmitted and Acquired HIV Type 1 Drug Resistance Mutations in the Swiss HIV Cohort Study From 1998 to 2012**. *J Infect Dis* 2015, **212**(1):28-38.

223. Ye JR, Lu HY, Wang WS, Guo L, Xin RL, Yu SQ, Wu TC, Zeng Y, He X: **The prevalence of drug resistance mutations among treatment-naive HIV-infected individuals in Beijing, China**. *AIDS Res Hum Retroviruses* 2012, **28**(4):418-423.

224. Yebra G, Holguin A, Pillay D, Hue S: **Phylogenetic and demographic characterization of HIV-1 transmission in Madrid, Spain**. *Infect Genet Evol* 2013, **14**(1):232-239.

225. Yebra GdM, M.; Pérez-Elías, MJ.; Pérez-Molina, JA.; Galán, JC.; Llenas-García, J.; Moreno, S.; Holguín, Á.: **Increase of transmitted drug resistance among HIV-infected sub-saharan africans residing in spain in contrast to the native population**. *PLoS ONE* 2011, **6**(10):no pagination.

226. Youmans E, Tripathi A, Albrecht H, Gibson JJ, Duffus WA: **Transmitted antiretroviral drug resistance in individuals with newly diagnosed HIV infection: South Carolina 2005-2009**. *South Med J* 2011, **104**(2):95-101.

227. Yu GL, Y.; Li, J.; Diao, L.; Yan, X.; Lin, P.; He, Q.; Wang, Y.; Fu, X.; Yang, F.; Long, Q.; Lin P.: **Genetic diversity and drug resistance of HIV type 1 circulating recombinant form-bc among drug users in Guangdong Province**. *AIDS Research and Human Retroviruses* 2009, **25**(9):869-875.

228. Zhang J, Guo Z, Pan X, Zhang W, Yang J, Ding X, Xu Y, Xia Y, Jiang J: **Highlighting the crucial role of Hangzhou in HIV-1 transmission among men who have sex with men in Zhejiang, China**. *Sci Rep* 2017, **7**(1):13892.

229. Zhang J, Guo Z, Yang J, Pan X, Jiang J, Ding X, Zhang W, Xia Y, Xu Y, Huang J: **Genetic diversity of HIV-1 and transmitted drug resistance among newly diagnosed individuals with HIV infection in Hangzhou, China**. *J Med Virol* 2015, **87**(10):1668-1676.

230. Zhang X, Li S, Li X, Li X, Xu J, Li D, Ruan Y, Xing H, Zhang X, Shao Y: **Characterization of HIV-1 subtypes and viral antiretroviral drug resistance in men who have sex with men in Beijing, China**. *AIDS* 2007, **21 Suppl 8**:S59-65.

231. Zhao B, Han X, Dai D, Liu J, Ding H, Xu J, Chu Z, Bice T, Diao Y, Shang H: **New trends of primary drug resistance among HIV type 1-infected men who have sex with men in Liaoning Province, China**. *AIDS Res Hum Retroviruses* 2011, **27**(10):1047-1053.

232. Zhao B, Han X, Xu J, Hu Q, Chu Z, Zhang J, Lu L, Wang Z, Fu J, Chen X *et al*: **Increase of RT-related transmitted drug resistance in non-CRF01_AE among HIV type 1-infected men who have sex with men in the 7 cities of China**. *J Acquir Immune Defic Syndr* 2015, **68**(3):250-255.

233. Zhong P, Kang L, Pan Q, Konings F, Burda S, Ma L, Xue Y, Zheng X, Jin Z, Nyambi P: **Identification and distribution of HIV type 1 genetic diversity and protease inhibitor resistance-associated mutations in Shanghai, P. R. China**. *J Acquir Immune Defic Syndr* 2003, **34**(1):91-101.

234. Zuckerman NS, Mor Z, Bucris E, Wax M, Mendelson E, Mor O: **Sexual intermingling of Arab and Jewish MSM in Israel: results of a molecular epidemiology study**. *AIDS* 2019, **33**(2):339-344.
