# Supplementary material for: Guidelines are needed for studies of pre-treatment HIV drug resistance: a methodological study
Source: BMC Med Res Methodol. 2021 Apr 19;21:76. doi: 10.1186/s12874-021-01258-1 (PMC8056637; doi:10.1186/s12874-021-01258-1)
Supplement: Supplementary file 4 — Additional file 4. Bar charts and scatterplots [file 12874_2021_1258_MOESM4_ESM.docx]

Additional figure 1: Mean number of items reported by region (n=229). Error bars are standard deviation.

Additional figure 2: Mean number of items reported by risk of bias (n=234). Error bars are standard deviation.

Additional figure 3: Mean number of items reported by source of funding (n=194). Error bars are standard deviation.

Additional figure 3: Mean number of items reported by income level (n=230). Error bars are standard deviation.

Additional figure 5: Mean number of items reported over time (n=234).

Additional figure 6: Number of items reported by sample size (n=234). Figure truncated at 10000 to improve scale (7 studies not shown).
